# Socially situated artificial intelligence enables learning from human interaction

**DOI:** 10.1073/pnas.2115730119

**Published:** 2022-09-19

**Authors:** Ranjay Krishna, Donsuk Lee, Li Fei-Fei, Michael S. Bernstein

**Affiliations:** ^a^Computer Science Department, Stanford University, Stanford, CA 94305

**Keywords:** human-centered AI, socially situated learning, computer vision, human–computer interaction

## Abstract

Regardless of how much data artificial intelligence agents have available, agents will inevitably encounter previously unseen situations in real-world deployments. Reacting to novel situations by acquiring new information from other people—socially situated learning—is a core faculty of human development. Unfortunately, socially situated learning remains an open challenge for artificial intelligence agents because they must learn how to interact with people to seek out the information that they lack. In this article, we formalize the task of socially situated artificial intelligence—agents that seek out new information through social interactions with people—as a reinforcement learning problem where the agent learns to identify meaningful and informative questions via rewards observed through social interaction. We manifest our framework as an interactive agent that learns how to ask natural language questions about photos as it broadens its visual intelligence on a large photo-sharing social network. Unlike active-learning methods, which implicitly assume that humans are oracles willing to answer any question, our agent adapts its behavior based on observed norms of which questions people are or are not interested to answer. Through an 8-mo deployment where our agent interacted with 236,000 social media users, our agent improved its performance at recognizing new visual information by 112%. A controlled field experiment confirmed that our agent outperformed an active-learning baseline by 25.6%. This work advances opportunities for continuously improving artificial intelligence (AI) agents that better respect norms in open social environments.

## Significance

Humans have long demonstrated an ability to learn from interactions with others. However, artificial intelligence (AI) agents learn in social isolation. To create intelligent systems that understand more than a fixed slice of the world, our article formalizes socially situated AI—a framework that enables agents to interact with people as they simultaneously learn new concepts about the world around them. Using our framework, we deploy a field experiment on a photo-sharing social network where our agent interacts with hundreds of thousands of people to learn concepts about the visual world. We combine advances in deep learning, computer vision, natural language processing, and human–computer Interaction to deliver a human-centered AI that learns from interactions with people in social environments.

Today’s methods for training artificial intelligence (AI) agents are akin to locking each agent alone in a room with a stack of books (1). Powered by large volumes of manually labeled training data (2, 3) or scraped web content (4, 5) for the agent to consume, machine learning has produced rapid progress in many tasks ranging from healthcare (6) to sustainability (7). But, when a concept is absent from the training data, the agent has no means to acquire it: Restricting an agent’s knowledge source to the books in the room prevents the agent from learning any concepts not present in the room. Worse, these methods ossify agents to ongoing changes to the world or to evolving human needs. So, while the resulting agents often demonstrate strong test set performance, they struggle when faced with novel situations or when deployed in the real world (8–10).

We present a formalization that enables AI agents to break out of the metaphorical room by learning through ongoing interactions with people in real-world social environments. We term this approach socially situated artificial intelligence and present evidence through a field experiment that it enables AI agents to learn new concepts that never occurred in their initial training data by simultaneously learning how to interact with people. Our method is inspired by human development, which is a socially mediated process in which children acquire new concepts and cultural norms through inquisitive dialogues with more knowledgeable members of society (11, 12). Enabling socially situated AI is critical to realizing many beneficent applications, especially in which effective human interactions are critical to improve an AI agent’s ability to understand and act accordingly, including human–computer interaction (13), interactive robotics (14), personalized conversational agents (15), and accessible technology (16).

To enable socially situated AI, the agent must not only gather data to learn new concepts, but also learn how to interact with people to gather the data. At any given moment, the agent must trade off between these twin goals of interacting to learn and learning to interact. The task is made more challenging because the space of possible interactions for the agent to traverse is vast, the space of useful social interactions is a sparse subset of these possible interactions, and the space of informative interactions constantly shifts as the agent learns. Reinforcement learning, which formalizes possible interactions as an action space and feedback as a reward, requires hundreds of millions of interactions to uncover this subspace of informative and prosocial interactions (17, 18); people will abandon such an agent long before it crosses such a threshold (19, 20). As a result of this limitation, methods that learn from human interaction have so far only seen success with manual human labels (21–25) or with small action spaces such as games and simulations with only a few dozen moves (26–28).

To overcome these challenges, we introduce a formalization of socially situated artificial intelligence as an iterative reinforcement learning problem where an agent seeks to improve an underlying model by interacting with people in a social environment where people may or may not respond informatively. Responses are useful only if they contain new information that is useful to the agent. The agent must thus choose social interactions that elicit new concepts useful for the model. Our formulation adopts a knowledge reward to guide the agent to interactions that lead to the discovery of new concepts and an interaction reward to guide the agent to interactions that adhere to social norms in the environment. New concepts are gathered from these interactions, which are used as training data to update the model. As the model improves at these concepts, the agent updates its policy and begins learning how to ask questions about new concepts where people are interested to answer but the model’s performance is still poor. This process of uncovering social norms, improving the underlying model, and updating the interaction policy iterates throughout the agent’s lifetime.

**Fig. 1.**
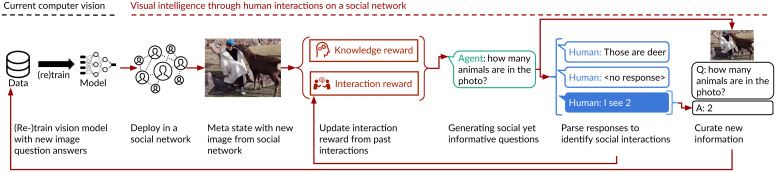
No matter how comprehensively we curate datasets, AI model deployments will inevitably encounter situations they have not previously seen, limiting their utility in the real world. We introduce a framework for socially situated AI, a reinforcement-learning framework that enables agents to uncover useful social interactions with people that result in the discovery of new information. With this formulation, we design a prototype to showcase the possibility of socially situated learning for a visual intelligence task. Our prototype agent learns visual concepts by asking questions about pictures people upload on social media. It parses how people respond to our agent into two rewards, which guide the agent toward interactions that are preferable for people and informative for its underlying model.

**Fig. 2.**
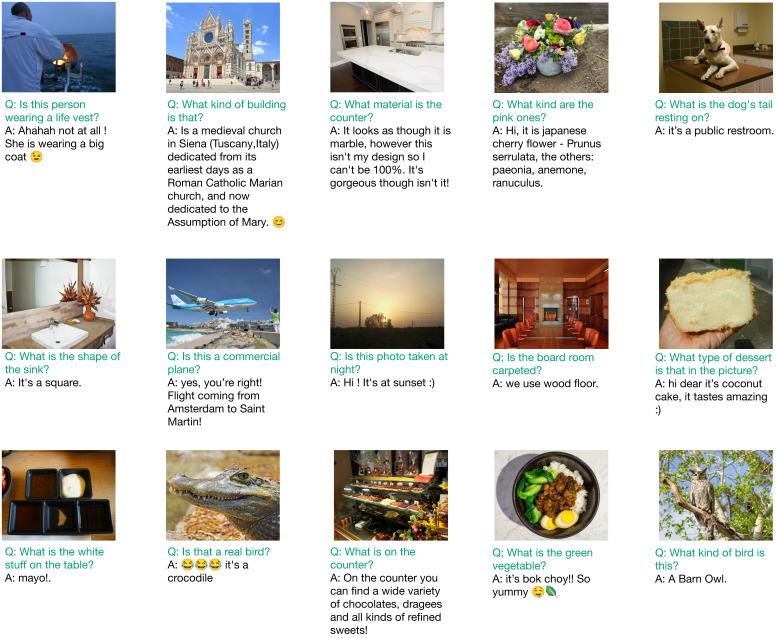
Examples of the 236,000 interactions initiated by our agent with people on social media, demonstrating the diversity of its questions and responses. Some questions verify concepts, while others ask about attributes such as types of buildings or materials of objects. The responses vary widely in length and vocabulary. While some directly answer our question, such as “It’s a square,” others provide a lot of background information. Some responses also indicate positive sentiment by using emojis, exclamations or phrases like “Hi dear.” Not shown here, each question is prepended with a self-disclosure introduction: “We are a computer science research project.” To preserve privacy, photos displayed are under a creative commons license and are visually and semantically similar to the ones uploaded by users on social media.

We explore the challenges associated with socially situated learning through a large-scale field experiment: We deploy an AI agent on a large photo-sharing social network to learn new visual concepts, a challenging task for which prior models have been criticized as being limited, brittle (29, 30), and prone to problematic behavior (31). Our agent interacts with people on social media by posting natural language questions as comments (Figs. 1 and 2). In a field experiment, we compare our agent to ablations that focus only on the knowledge reward (traditional active learning) or only on the interaction reward. From 236,000 interactions, the one agent using both is capable of learning and dramatically improves its visual intelligence while the control variants stop receiving feedback or quickly stop learning.

## Socially Situated AI Framework

Active learning is the most common framework consulted when iteratively expanding a model’s capabilities. The goal of active learning is to optimize a sequence of labeling requests to acquire new data D; the new data will be used to improve performance on the model V:X→Y with as few requests as possible. Although most active-learning methods design heuristic acquisition algorithms (32), recent work has formalized the process as a reinforcement-learning process (25). These attempts usually remove real humans from the pipeline and assume the existence of an oracle that will provide labels for any request.

Although a pure active-learning approach could gather new data through social interactions in social environments, recent work in human–computer interaction has concluded that users do not want to serve as simple oracles by repeatedly providing labels, breaking a fundamental assumption in active learning (33–35). Our work is a reaction to this observation: Traditional active learning is not ecologically valid in realistic social environments. In our field experiments, we empirically show that a baseline active-learning approach generates interactions that people are not interested in responding to (25).

We formalize socially situated artificial intelligence as an iterative reinforcement-learning problem that generalizes conventional active learning. The agent is placed in a social environment $E=(S,A,P,P_{0})$. S is the state of environment; e.g., it could include the history of dialogues for a conversational agent or the current location of objects in a three-dimensional world for a robotic agent. A is the space of possible interactions with people that the agent can initiate; e.g., it can be the set of statements that a conversational agent can ask or the set of motions a robotics agent can perform. P:S×A→S is the transition dynamics; e.g., the transition function encodes how people react to the agent’s past actions and how the world changes as a result. Finally, $P_{0}$ is the probability measure on the initial state distribution.

Similar to active learning, the agent’s goal is to gather data D to optimize V’s performance with as few interactions as possible. We design this agent’s decision-making process as an infinite-horizon Markov decision process $M=(\bar{S},A,\bar{P},{\bar{P}}_{0},R)$. Intuitively, M jointly characterizes the evolution of the environment E, collected data history D, and the model V as the agent makes interaction decisions to optimize the learning objective encoded by the reward R. $\bar{S}=(S\times D\times V)$ is a metastate that now encodes the state of the environment S, the data history D, and the current capabilities of the underlying model V. So, at a given time step, ${\bar{s}}_{t}\in \bar{S}=(s_{t},D_{t},V_{t})$, where $D_{t}=\{s_{0},a_{0},\ldots ,s_{t}\}\in D$ is the dataset of past interactions. *D_t_* is a raw form of the data collected so far and can be postprocessed to yield training data for $V_{t}$. The metatransition dynamics are $\bar{P}:\bar{S}\times A\to \bar{S}$ such that $s_{t+1}\sim P(\cdot |s_{t},a_{t})$, new interactions are added to the dataset $D_{t+1}=D_{t}\;\cup \;\{a_{t},s_{t+1}\}$, and a new model is trained from the accumulated data $V_{t+1}=\text{train}(D_{t+1})$. The metainitial state distribution is ${\bar{P}}_{0}(\bar{s}=(s,D_{0},V_{0}))=P(s)\cdot 1[D_{0}=\{\}]\cdot \text{init}(V)$, where init(V) is the prior distribution over $V_{0}$ initialized parameters and *D*_0_ is an empty dataset.

To make socially situated learning possible, we design rewards that balance the twin goals of interacting to learn and learning to interact. We design the reward $R:\bar{S}\to R=\alpha \cdot R_{\text{interaction}}+(1-\alpha )\cdot R_{\text{knowledge}},0\leq \alpha \leq 1$, to be a linear combination of two rewards: an interaction reward and a knowledge reward. The interaction reward encourages interactions within the community’s prosocial norms. Unlike prior work (21, 24, 28), we do not assume that people can be trained to provide explicit rewards or assume that people would respond to every interaction with useful information (25, 32). Instead, by drawing on the concept of nonreactive measures from sociology (36, 37), which suggests that humans learn social norms or interaction preferences by observing how people in a community interact with them, our agent scalably learns social norms established within the social environment E, not through repeated interactions with one person but through a single interaction with hundreds of thousands of people within the environment. The knowledge reward encourages interactions that result in data that maximally improve the performance of V. For example, it can be modeled as an active-learning acquisition function (32) with respect to V.

The socially situated agent learns a policy $\pi :\bar{S}\to A$ that maps from the current metastate to interactions. When interacting with modalities such as language and motion, the spaces of possible sentences and gestures are combinatorially vast; domain-specific methods would need to develop techniques to make exploring the space of A tractable. The optimal policy maximizes the rewards: $\pi^{*}=\arg\max_{\pi }E_{\pi }[\sum_{t}R({\bar{s}}_{t})]$. We evaluate our agent’s performance using two metrics. First, borrowing from active learning, we report accuracy of the underlying model V on a held-out test set of $(X,Y)\in D_{\text{test}}$. Second, given the prior human–computer interaction literature arguing that people will disengage when they are not interested (38–41), we evaluate whether our agent learns appropriate social interactions using the rate of informative interactions: the percentage of the agent’s interactions that resulted in new information. A higher informative response rate implies a greater understanding of implicit social norms while a low informative response rate implies people are not responding, which can slow down or even halt the socially situated learning process.

By appropriately constructing M, we can recover different variants of traditional active learning (*SI Appendix*, section 1). Prior work sets *α* to zero, making the assumption of an oracle that will generate new information for any query or interaction. In contrast, by extending the framework as a reinforcement-learning problem that characterizes social interactions within the reward $R_{\text{interaction}}$, we explicitly model human-interaction preferences and empirically show that it is necessary for socially situated learning. Aside from active learning, this formulation also generalizes recent work on social learning (42, 43) and machine theory of mind (44). By designing the interaction reward as a dynamics model to predict how others will act, we recover these related formulations. Intuitively, the formulation affords a foundation that can be applied to applications such as conversational assistants, teachable agents in education, and assistive robotics (45).

## Visual Intelligence through Human Interactions

In this section, we apply the socially situated AI framework to computer vision using a photo-sharing social network. Modern computer vision systems rely on large volumes of human-labeled training data, but generating these datasets remains challenging. In computer vision, for example, the ImageNet dataset (46) required 14 million labels such as whether an image contains a chair. Unfortunately, this knowledge is both so simple that it is extremely tedious for humans to label and so tacit that it is often absent from the image’s metadata and the human annotators are required. This combination of tedious and tacit makes computer vision data challenging to acquire; many volunteer data acquisition efforts fail (47, 48), limiting the scale and ambition of visual intelligence efforts.

To explore the potential of harnessing this tacit information through social interactions, we empirically validate the utility of the framework by deploying a socially situated agent on Instagram, a large photo-sharing social network (see *SI Appendix*, section 2 about our Institutional Review Board [IRB]). The agent interacts with people by asking natural language questions $q_{t}\in A$; i.e., A is the space of possible language interactions. The state $s_{t}=(i_{t},ans_{t})$ encodes a randomly sampled new image *i_t_* uploaded to the social network and the human answer *ans_t_* to the agent’s previous question $q_{t-1}$. The answer *ans_t_* can be an empty string if there is no response. The answers are extracted from human responses using a natural language parser (see *SI Appendix* section 7 on parsing responses).

The agent uses the answers extracted from human responses to learn from its interactions. Using natural language to gather visual knowledge allows us to test a whole host of common computer vision recognition tasks: object detection (e.g., “What is in the image?”), fine-grained recognition (e.g., “What kind of flowers are in the vase?”), attribute classification (e.g., “What material is the table made of?”), knowledge base reasoning (e.g., “Is this a food vegetarian?”), and commonsense reasoning (e.g., “Was this taken in the winter?”). Consequently, we design V to be a computer vision question answering model. The inputs $(i_{t},q_{t})\in X$ are an image and corresponding natural language question and the output is $ans_{t}\in Y$, a natural language answer. The agent’s goal is to improve V’s ability to recognize visual concepts (see *SI Appendix*, section 3 discussion #2 for more details). From its interactions, the acquired dataset $D_{t}=\{(i_{0},q_{0},ans_{0}),\ldots ,(i_{t-1},q_{t-1},ans_{t-1})\}$ is used to train $V_{t}$. Drawing on the active-learning literature, we design the knowledge reward $R_{\text{knowledge}}$ as V’s uncertainty. Initially the recognition model does not know how to recognize any concepts but becomes more confident the more often it sees a particular concept. For example, the recognition model might not know how to recognize deer, resulting in high uncertainty whenever it encounters one. If people identify the animal for the agent, its uncertainty will decrease, guiding its behavior to ask about other concepts.

The agent uses its past interactions to learn how to interact. Social science observes that each community develops distinct norms and cultures, influencing how people interact with one another (49, 50). On social networks, for instance, people prefer answering shorter requests, providing factual knowledge, and avoid rhetorical requests or vague questions (51). Every past interaction is marked as either a positive (interactions that result in new information) or a negative example (interactions that receive no new information). These positive and negative examples are used to continually train $R_{\text{interaction}}:S\times A\to [0,1]$ as a binary classifier. This interaction reward guides the agent’s behavior toward the community’s prosocial norms.

Finding useful language interactions is a combinatorial search problem; we draw on recent advances in machine learning to tractably explore the combinatorial interaction space. A straightfoward approach could devise the agent’s policy as an image-to-question generation model, $q_{t}\sim \pi (i_{t})$, where *q_t_* is composed of a sequence of words. This combinatorial search process needs to be iteratively repeated; as V’s capabilities improve, the space of informative interactions continuously shifts. To make this search process tractable, we apply an observation from several intersecting social science fields: While the action space of all possible behaviors is vast, most human–human interactions lie on a low-dimensional space (52). For example, language use is Zipf distributed (53), norms and social scripts encourage common behaviors within groups (54, 55), and cultures develop preferred interrelated emblem gestures (56). We learn an interaction representation of realistic human–human interactions using existing literature in information maximizing variational autoencoders (57, 58). We use this representation, *z*, as an intermediate constraint by reconfiguring the policy, $z_{t}\sim \pi_{\theta }(i_{t})$, to project the input image *i_t_* into the representation space and by designing a decoder, $q_{t}\sim dec_{\phi }(z)$, to project from the representation to a sequence of words. *θ* and ϕ parameterize the neural networks used to define the policy and the decoder. Once trained, the decoder parameters, ϕ, are held constant throughout deployment, decoupling the agent’s need to concurrently learn what interactions to initiate with how to generate those interactions. Rewards can be assigned to a single action (*z*) rather than a subset of the words in the question (see *SI Appendix*, Section 3 for more details).

## Field Experiment

The socially situated AI framework sets up two simultaneous goals for the agent: one to initiate social interactions that people want to respond to with informative data and another to improve its underlying model by gathering useful data. These two goals define the evaluation metrics we use to evaluate the deployed agent. First, to evaluate the agent’s ability to garner responses, we measure the rate of informative responses to its questions (38–41), i.e., the percentage of the agent’s interactions that received an answer. We detect informative responses using a response model that identifies whether the response contains an answer. Second, to evaluate our agent’s ability to recognize new visual concepts, we report accuracy of the knowledge reward’s recognition model on a held-out test set of 50,104 social media images, questions, and answers, collected using annotators from Amazon Mechanical Turk (*SI Appendix*, section 10).

To compare the socially situated agent designed using our framework versus others, we also deploy a baseline approach and two ablations of our agent. To isolate the effects of both the interaction and the knowledge rewards, we deploy the human preference ablation, which uses only the interaction reward, and the active-learning ablation, which uses only the knowledge reward. The active-learning ablation is a reinforcement-based active learning introduced in prior work (25). We also deploy a baseline agent that does not use the pretrained interaction representation as the action space and is allowed to fine tune the decoder’s parameters, allowing the agent to use the entire combinatorial vocabulary space as the action space (59). This baseline uses both the rewards; we also add an extra language-modeling reward, which encourages the generation of grammatically correct language outputs and is used in prior literature (60). All agents are trained using proximal policy gradients (61). All agents are initialized using the same amount of data and have the same policy and decoder architectures. The agent self-identified as an AI research project, and workers on Amazon Mechanical Turk vetted all questions prior to posting to ensure that none would be problematic or offensive (see *SI Appendix*, section 8 for the vetting task workflow).

**Fig. 3.**
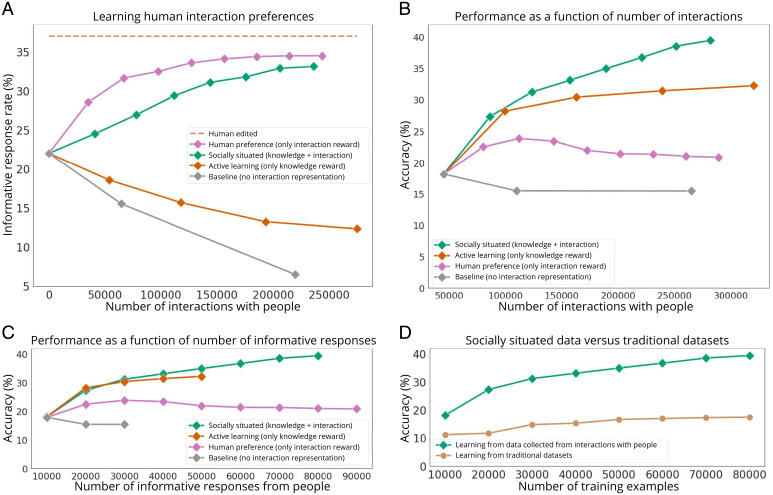
We show changes in informative response rate and recognition accuracy as agents interact with people and gather new visual knowledge. (*A*) We plot informative response rate versus the number of interactions initiated. Socially situated and human preference agents, which use the interaction reward, increase the likelihood of answers from people. Other agents observe a decrease in responses, stunting their data acquisition. (*B*) We visualize the performance of the vision model versus the number of interactions initiated by the agent. Socially situated and active-learning agents, which use the knowledge reward, gather useful data, with active learning requiring more interactions and plateauing since fewer people are responding. (*C*) We visualize the performance of the vision model versus the number of responses from people. Even though socially situated is trading off the two rewards, its accuracy improvements are on par with active learning, which solely maximizes the knowledge reward. (*D*) We report recognition accuracy as a function of number of training examples from our socially collected data versus traditional datasets, demonstrating that socially situated agents can in fact acquire new information that is not present in traditional datasets.

Through a deployment of 8 mo where each agent was allowed to initiate at least 200,000 interactions, we observe the benefits of socially situated learning over other methods (Fig. 3*A*). The socially situated agent increases its informative response rate from an initial 22% to 33% across 236,000 interactions, a relative improvement of 50%. In comparison, both the active-learning and baseline agents elicit fewer responses after every iteration, ending at 6% and 12.3%, respectively. The baseline agent, in its effort to explore the combinatorial space of all possible language interactions, inevitably generates incoherent questions, causing response rates to decrease; this produces a vicious cycle where the agent cannot identify useful interactions. We halted its deployment once response rates dropped to 6%. The active-learning agent generates longer and harder questions that people are not interested in answering. For example, some of its questions require external knowledge: “Are these tools designed for someone left handed or right handed?” requires knowledge about a specific tool and whether it can be operated by either hand. The human preference agent achieves the highest response rate but reduces the requests to a small set that people prefer answering. It asks easy-to-answer questions, for example those that are time related (e.g., “What time of the day was this picture taken?”) and color related (e.g., “What color is the shirt?”).

We also perform an experiment where we hire human annotators to edit our questions to increase the likelihood of responses. This human-edited experiment, which achieves 37% responses, measures the average human ability to elicit responses from our chosen social environment assuming that the original intention of the question remains the same. This value measures how much more data could be collected (i.e., how much the informative response rate could be increased) if the AI agent had more social capability. This value is slightly higher than the agent’s final performance of 33%. We also study how self-identifying as an AI agent impacts the response rate and find no statistical difference, suggesting that people’s responses are not a reaction to the novelty or citizen science motivation to help a research project succeed (*SI Appendix*, section 14).

Using the data collected to improve the computer vision model, the socially situated agent improves accuracy using fewer interactions than other agents. It achieves a model performance of 39.44% within 236,000 (Fig. 3*B*) interactions, from which it receives 70,000 responses (Fig. 3*C*). Meanwhile, active learning initiates 274,893 interactions to receive only 30,000 responses and begins to saturate performance at 31.4%; with the response rate drop to 12.3%, these results empirically suggest that a pure active-learning approach is not ecologically viable in some social environments. Even though the human preference agent receives more responses per interaction, the data it collects do not improve the underlying vision model. Since it favors collecting answers to the same small set of questions, the underlying vision model begins to overfit to generating only time-related and color-related outputs. Finally, since the baseline agent begins to generate incoherent questions, the data it collects are not useful.

We further compare how training using data collected by the socially situated agent compares against that using data from existing datasets (62, 63) (Fig. 3*D*). Training with the same number of labels from existing datasets increases accuracy only from 11.24% to 17.45%, vs. an increase from 18.13% to 39.44% when training with our socially situated data, demonstrating that socially situated agents are capable of adapting to the distribution of data encountered in their social environment.

**Fig. 4.**
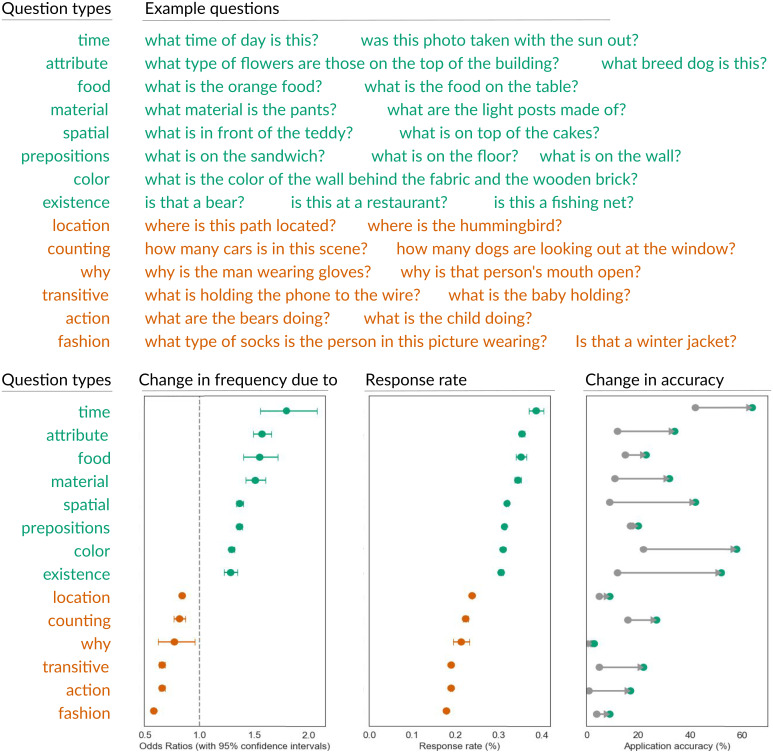
Our agent emergent behavior is consistent with social science literature on how people behave online. We group the generated questions into categories and visualize the change in odds ratio of generating questions in that category, the response rate, and the change in recognition accuracy.

To examine what social norms were learned, we manually group questions into 21 categories (Fig. 4) (57). Using a logistic regression, we report the log odds of our agent asking questions from each category at the beginning versus at the end of its deployment. Our agent’s learned behavior is consistent with human learned behavior (39, 49, 51): It produces more tailored questions in categories that can be easily interpreted and answered (50), such as materials (e.g., “What are the light posts made of?”), existence (e.g., “Is that a bear?”), and color (e.g., “What color is that vase?”); it reduces the generation of questions that require effort to answer or have multiple answers (49, 51), such as counting (e.g., “How many cars are in the scene?”), actions (e.g., “What is the child doing?”), and why (“Why is the man wearing gloves?”). It learns to demonstrate social proof by mentioning recognizable concepts (39), such as spatial (e.g., “What is in front of the teddy?”), prepositions (e.g., “What is on the wall?”), and attributes (e.g., “What type of flowers are those on top of the building?”).

## Conclusion

Our work presents a framework that relocates AI agents outside of the metaphorical locked room of their training data and enables ongoing collaborative learning with people. Agents can guide their learning by interacting with real people in online social environments. Our framework breaks the ecologically invalid assumption that oracles will always provide new concepts and expects machines to socially engage in interactions to learn from people. Our field study demonstrated that socially situated learning is possible on large combinatorial interaction domains such as natural language question generation, enabling agents to discover new concepts and simultaneously uncover social norms. Success required the development of design patterns to ensure prosocial interactions: The agent self-identified as a research project; we hired crowd workers to continually monitor and prevent accidental antisocial agent behavior; we discontinued the baseline agents when they began producing nonsensical interactions (*SI Appendix*, section 15 on ethics). While we illustrate our framework through a visual intelligence application using language interactions, we believe that this work more broadly advances opportunities for AI agents to interact as collaborative partners—with applications including healthcare support robots that can ask providers to clarify procedures, technologies that can improve their interfaces through user feedback, and agents that can learn from many different communities to diversify their understanding.

## Materials and Methods

### Informed-Consent Procedures.

Our research was approved (protocol 50287) by Stanford University’s IRB through an expedited nonmedical review. Our IRB approves data collection from two online population pools: one from workers on Amazon Mechanical Turk and another from users worldwide on a social network.

We poll images from a social network, generate questions about concepts in the image, and ask social network users by posting the question on their posted image. The questions are programmatically generated and vetted by Amazon Mechanical Turk workers as not being problematic or offensive. Only questions that are approved by workers are posted online to users.

Mechanical Turk workers are fully informed about the purpose of the study. They are told that we plan to generate questions that would fit the social norms within the community and would be likely to receive an answer from an online social network user. Since our questions are automatically generated, workers are asked to identify questions that might be construed as offensive or rude to ask. They are informed that all questions that are vetted will be posted on social media. They are shown the image associated with the question but are not provided with links to the social network post or the poster’s account.

Social network users are informed that we are asking a question about their image. All questions are preceded by the following introduction: “We are a computer science research project.” The social network profile used to post the question also has the same message printed as its biography. Regardless of whether users respond, we debrief them of their participation by sending them a direct message on the social network after 48 h of posting the question. We provide them with an email address in case they have further questions or reservations: “Thank you for responding to our question. Your answers will be used to improve an AI agent’s ability to recognize concepts in images. Your original image and answer will not be released publicly. If you wish that we do not use your response or have questions about the study, please email us at <EMAIL_ADDRESS > or reply to this message.”

### Data Privacy.

We collect worker IDs from Mechanical Turk workers (which are anonymized). We also collect usernames for social media participants, which are publicly available (however, usernames, personal information, etc., will not be used for any experiments or stored). Data are transferred using secured folders on Stanford University’s file system. Since our primary contribution is a framework and a proof-of-concept prototype, the data we collect will not be shared publicly. Participants are contacted by us only if their posts are publicly accessible. We collect only publicly available data.

## Supplementary Material

Supplementary File

## Data Availability

Anonymized (images and text) data have been deposited in https://github.com/stanfordvl/ssai.git (64) and https://doi.org/10.5281/zenodo.6878328 (65). All other study data are included in this article and/or supporting information. Our contribution is a framework for machine learning agents to learn from interactions with humans, and not a dataset. Following the guidelines set in our IRB, we are unable to release user images and user-generated questions as they may contain private information about the users. *SI Appendix* section 14 discusses the risks of releasing such data and proposes directions for future research work to safely release user generated data.

## References

[1] F. Jackson, Epiphenomenal qualia. Philos. Q. 32, 127–136 (1982).

[2] A. Krizhevsky, I. Sutskever, G. E. Hinton, “Imagenet classification with deep convolutional neural networks” in Advances in Neural Information Processing Systems, P. L. Bartlett, F. C. N. Pereira, C. J. C. Burgess, L. Bottou, K. Q. Weinberger, Eds. (Lake Tahoe, NV, 2012), pp. 1106–1114.

[3] M. L. Gray, S. Suri, Ghost Work: How to Stop Silicon Valley from Building a New Global Underclass (Eamon Dolan Books, 2019).

[4] T. Mitchell ., Never-ending learning. Commun. ACM 61, 103–115 (2018).

[5] T. B. Brown ., “Language models are few-shot learners” in Advances in Neural Information Processing Systems, I. Guyon ., Eds. (Long Beach, CA, 2020), pp. 4299–4307.

[6] A. Haque, A. Milstein, L. Fei-Fei, Illuminating the dark spaces of healthcare with ambient intelligence. Nature 585, 193–202 (2020). 32908264 10.1038/s41586-020-2669-y

[7] N. Jean ., Combining satellite imagery and machine learning to predict poverty. Science 353, 790–794 (2016). 27540167 10.1126/science.aaf7894

[8] G. Marcus, E. Davis, Rebooting AI: Building Artificial Intelligence We can Trust (Pantheon, 2019).

[9] G. Marcus, Deep learning: A critical appraisal. *arXiv* [Preprint] 13 August 2018.

[10] T. Winograd, Thinking Machines: Can There Be? Are We? (Department of Computer Science, Stanford University, 1987).

[11] L. S. Vygotski, Thought and Language (MIT Press, 1962).

[12] J. Newson, The growth of shared understandings between infant and caregiver. (Cambridge University Press, Cambridge, United Kingdom, 1979), pp. 207–222.

[13] E. Horvitz, “Principles of mixed-initiative user interfaces” in *Proceedings of the SIGCHI Conference on Human Factors in Computing Systems*, M. G. Williams, M. W. Altom, Eds. (ACM, Pittsburgh, PA 1999), pp. 159–166.

[14] A. D. Dragan, K. C. Lee, S. S. Srinivasa, “Legibility and predictability of robot motion” in *2013 8th ACM/IEEE International Conference on Human-Robot Interaction (HRI)*, H. Kuzuoka, V. Evers, M. Imai, J. Forlizzi, Eds. (Tokyo, Japan, 2013), pp. 301–308.

[15] J. Grudin, R. Jacques, “Chatbots, humbots, and the quest for artificial general intelligence” in *Proceedings of the 2019 CHI Conference on Human Factors in Computing Systems, CHI ’19*, S. A. Brewster, G. Fitzpatrick, A. L. Cox, V. Kostakos, Eds. (ACM, New York, NY, 2019), pp. 209:1–209:11.

[16] M. R. Morris, Ai and accessibility. Commun. ACM 63, 35–37 (2020).

[17] M. Lewis, D. Yarats, Y. Dauphin, D. Parikh, D. Batra, “Deal or no deal? End-to-end learning of negotiation dialogues” in Proceedings of the 2017 Conference on Empirical Methods in Natural Language Processing, M. Palmer, R. Hwa, S. Riedel, Eds. (Copenhagen, Denmark, 2017), pp. 2443–2453.

[18] J. Li ., “Deep reinforcement learning for dialogue generation” in Proceedings of the 2016 Conference on Empirical Methods in Natural Language Processing (EMNLP), J. Su, X. Carreras, K. Duh, Eds. (Austin, TX, 2016), pp. 1192–1202.

[19] H. Y. Shum, X. He, D. Li, From eliza to xiaoice: Challenges and opportunities with social chatbots. Front. Inf. Technol. Electron. Eng. 19, 10–26 (2018).

[20] E. Luger, A. Sellen, “Like having a really bad PA: The gulf between user expectation and experience of conversational agents” in Proceedings of the 2016 CHI Conference on Human Factors in Computing Systems, J. Kaye, A. Druin, C. Lampe, D. Morris, J. P. Hourcade, Eds. (ACM, 2016), pp. 5286–5297.

[21] A. L. Thomaz, C. Breazeal, Reinforcement Learning with Human Teachers: Evidence of Feedback and Guidance with Implications for Learning Performance (Aaai, Boston, MA, 2006), vol. 6, pp. 1000–1005.

[22] A. L. Thomaz, C. Breazeal, Teachable robots: Understanding human teaching behavior to build more effective robot learners. Artif. Intell. 172, 716–737 (2008).

[23] M. Cakmak, A. L. Thomaz, “Designing robot learners that ask good questions” in *Proceedings of the* Seventh Annual *ACM/IEEE* International Conference *on Human-Robot Interaction*, H. A. Yanco, A. Steinfeld, V. Evers, O. Chadwicke Jenkins, Eds. (ACM, Boston, MA, 2012), pp. 17–24.

[24] C. L. Isbell ., Cobot in lambdamoo: An adaptive social statistics agent. Auton. Agent. Multi Agent Syst. 13, 327–354 (2006).

[25] I. Misra ., “Learning by asking questions” in 2018 IEEE/CVF Conference on Computer Vision and Pattern Recognition (IEEE, Salt Lake City, UT, 2018), pp. 11–20.

[26] P. F. Christiano ., “Deep reinforcement learning from human preferences” in Advances in Neural Information Processing Systems, I. Guyon ., Eds. (Long Beach, CA, 2017), pp. 4299–4307.

[27] D. Silver ., Mastering the game of go with deep neural networks and tree search. Nature 529, 484–489 (2016). 26819042 10.1038/nature16961

[28] C. L. Breazeal, “Sociable machines: Expressive social exchange between humans and robots,” PhD thesis, Massachusetts Institute of Technology, Cambridge, MA (2000).

[29] K. Eykholt ., “Robust physical-world attacks on deep learning visual classification” in *Proceedings of the IEEE Conference on Computer Vision and Pattern Recognition* (Salt Lake City, UT, 2018), pp. 1625–1634.

[30] A. Athalye, L. Engstrom, A. Ilyas, K. Kwok, “Synthesizing robust adversarial examples” in *International Conference on Machine Learning*, J. G. Dy, A. Krause, Eds. (PMLR, Stockholm, Sweden, 2018), pp. 284–293.

[31] G. Neff, P. Nagy, Talking to bots: Symbiotic agency and the case of Tay. Int. J. Commun. 10, 17 (2016).

[32] B. Settles, “Active learning literature survey” (Tech. Rep., University of Wisconsin-Madison Department of Computer Sciences, Madison, WI (Morgan & Claypool, 2009).

[33] M. Cakmak, C. Chao, A. L. Thomaz, Designing interactions for robot active learners. IEEE Trans. Auton. Ment. Dev. 2, 108–118 (2010).

[34] M. Cakmak, A. L. Thomaz, “Optimality of human teachers for robot learners” in 2010 IEEE 9th International Conference on Development and Learning, B. Kuipers, T. R. Shultz, A. Stoytchev, C. Yu, Eds. (IEEE, Ann Arbor, MI, 2010), pp. 64–69.

[35] A. Guillory, J. A. Bilmes, “Simultaneous learning and covering with adversarial noise” in *ICML* (2011), vol. 11, pp. 369–376.

[36] Z. Zhao ., “Recommending what video to watch next: A multitask ranking system” in *Proceedings of the 13th ACM Conference on Recommender Systems* (2019), pp. 43–51.

[37] E. J. Webb, D. T. Campbell, R. D. Schwartz, L. Sechrest, Unobtrusive Measures (Sage Publications, 1999), vol. 2.

[38] L. Von Ahn, L. Dabbish, “Labeling images with a computer game” in *Proceedings of the SIGCHI Conference on Human Factors in Computing Systems* (ACM, 2004), pp. 319–326.

[39] R. B. Cialdini, Influence: The Psychology of Persuasion, vol. 3 (A. Michel Port Harcourt, 1987).

[40] M. Dontcheva, R. R. Morris, J. R. Brandt, E. M. Gerber, “Combining crowdsourcing and learning to improve engagement and performance” in *Proceedings of the SIGCHI Conference on Human Factors in Computing Systems*, M. Jones, P. A. Palanque, A. Schmidt, T. Grossman, Eds. (ACM, Toronto, ON, Canada, 2014), pp. 3379–3388.

[41] P. G. Ipeirotis, E. Gabrilovich, “Quizz: Targeted crowdsourcing with a billion (potential) users” in *Proceedings of the 23rd International Conference on World Wide Web*, C.-W. Chung, A. Z. Broder, K. Shim, T. Suel, Eds. (ACM, Seoul, Republic of Korea, 2014), pp. 143–154.

[42] K. K. Ndousse, D. Eck, S. Levine, N. Jaques, “Emergent social learning via multi-agent reinforcement learning” in *International Conference on Machine Learning*, Vol 139, M. Meila, T. Zhang, Eds. (PMLR, 2021), pp. 7991–8004.

[43] N. Jaques ., “Social influence as intrinsic motivation for multi-agent deep reinforcement learning” in *International Conference on Machine Learning* (PMLR), P. A. Ortega, D. J. Strouse, J. Z. Leibo, N. de Freitas, Eds. (Long Beach, CA, 2019), pp. 3040–3049.

[44] N. Rabinowitz ., “Machine theory of mind” in *International Conference on Machine Learning* (PMLR), J. G. Dy, A. Krause, Eds. (Stockholm, Sweden, 2018), pp. 4215–4224.

[45] I. Serban, A. Sordoni, Y. Bengio, A. Courville, J. Pineau, “Building end-to-end dialogue systems using generative hierarchical neural network models” in *Proceedings of the AAAI Conference on Artificial Intelligence*, D. Schurrmans, M. P. Wellman, Eds. (AAAI Press, Phoenix, AZ, 2016), vol. 30, pp. 3776–3784.

[46] J. Deng ., “Imagenet: A large-scale hierarchical image database” in IEEE Conference on Computer Vision and Pattern Recognition (IEEE, Miami, FL, 2009), pp. 248–255.

[47] B. M. Hill, “Almost Wikipedia: Eight early encyclopedia projects and the mechanisms of collective action” in Essays on Volunteer Mobilization in Peer Production (Tech. Rep., Massachusetts Institute of Technology, Cambridge, MA, 2013), pp. 1–38.

[48] J. Reich, R. Murnane, J. Willett, The state of Wiki usage in US K–12 schools: Leveraging web 2.0 data warehouses to assess quality and equity in online learning environments. Educ. Res. 41, 7–15 (2012).

[49] R. E. Kraut, P. Resnick, Encouraging contribution to online communities in *Building successful online communities: Evidence-based social design.* (MIT Press, Cambridge, MA, 2011), pp. 21–76.

[50] K. R. Lakhani, E. Von Hippel, “How open source software works:“Free” user-to-user assistance” in Produktentwicklung mit Virtuellen Communities, C. Herstatt, J. G. Sander, Eds. (Springer, 2004), pp. 303–339.

[51] M. R. Morris, J. Teevan, K. Panovich, “What do people ask their social networks, and why? A survey study of status message q&a behavior” in *Proceedings of the SIGCHI Conference on Human Factors in Computing Systems*, E. D. Mynatt ., Eds. (Atlanta, GA, 2010), pp. 1739–1748.

[52] H. Narayanan, S. Mitter, “Sample complexity of testing the manifold hypothesis” in Advances in Neural Information Processing Systems, J. D. Lafferty, C. K. I. Williams, J. S. Taylor, R. S. Zemel, A. Culotta, Eds. (Vancouver, BC, Canada, 2010), pp. 1786–1794.

[53] G. K. Zipf, Human Behavior and the Principle of Least Effort (Cambridge University Press, 1949).

[54] R. C. Schank, R. P. Abelson, Scripts, Plans, Goals, and Understanding: An Inquiry into Human Knowledge Structures (Psychology Press, 2013).

[55] E. Fehr, U. Fischbacher, Social norms and human cooperation. Trends Cogn. Sci. 8, 185–190 (2004). 15050515 10.1016/j.tics.2004.02.007

[56] A. Kendon, Gesture: Visible Action as Utterance (Cambridge University Press, 2004).

[57] R. Krishna, M. Bernstein, L. Fei-Fei, “Information maximizing visual question generation” in IEEE Conference on Computer Vision and Pattern Recognition (Long Beach, CA, 2019).

[58] A. van den Oord, O. Vinyals, K. Kavukcuoglu, “Neural discrete representation learning” in Advances in Neural Information Processing Systems, I. Guyon ., Eds. (Long Beach, CA, 2017), pp. 6306–6315.

[59] J. Li, A. H. Miller, S. Chopra, M. Ranzato, J. Weston, “Learning through dialogue interactions by asking questions” in International Conference on Learning and Representation (Toulon, France, 2017).

[60] T. Shen, A. Kar, S. Fidler, “Learning to caption images through a lifetime by asking questions” in *Proceedings of the IEEE International Conference on Computer Vision* (Seoul, Republic of Korea, 2019), pp. 10393–10401.

[61] J. Schulman, F. Wolski, P. Dhariwal, A. Radford, O. Klimov, Proximal policy optimization algorithms. *arXiv* [Preprint] 13 August 2018.

[62] S. Antol ., “Vqa: Visual question answering” in *Proceedings of the IEEE International Conference on Computer Vision* (Santiago, Chile, 2015), pp. 2425–2433.

[63] R. Krishna ., Visual genome: Connecting language and vision using crowdsourced dense image annotations. Int. J. Comput. Vis. 123, 32–73 (2017).

[64] R. Krishna, D. Lee, L. Fei-Fei, M. Bernstein, Learning to Interact and Interacting to Learn with Socially Situated Artificial Intelligence. GitHub. https://github.com/stanfordvl/ssai.git. Deposited 19 July 2022.

[65] R. Krishna, Data from “Socially situated artificial intelligence enables learning from human interaction.” Zenodo. 10.5281/zenodo.6878328. Deposited 21 July 2022. PMC9522339 36122244

