# Supplementary material for: Socially situated artificial intelligence enables learning from human interaction
Source: Proc Natl Acad Sci U S A. 2022 Sep 19;119(39):e2115730119. doi: 10.1073/pnas.2115730119 (PMC9522339; doi:10.1073/pnas.2115730119)
Supplement: Supplementary File [file pnas.2115730119.sapp.pdf]

PNAS
www.pnas.org

Supplementary Information for

Socially Situated Artificial Intelligence Enables Learning from Human Interaction

Ranjay Krishna, Donsuk Lee, Li Fei-Fei*, Michael Bernstein*

Ranjay Krishna.
E-mail: ranjaykrishna@cs.stanford.edu

This PDF file includes:

Supplementary text
Figs. S1 to S9 (not allowed for Brief Reports)
Tables S1 to S2 (not allowed for Brief Reports)
SI References

Supporting Information Text

1. Recovering conventional active learning from Social Situated AI

Our socially situated learning generalizes different variants of conventional active learning methods.

- For example, **query membership synthesis active learning** (1), the agent queries for a label for an input $x \in \mathcal{X}$ and receives a label $y \in \mathcal{Y}$ as a response. So, $\mathcal{A} = \mathcal{X}$, $\mathcal{S} = \mathcal{Y}$ and the transition dynamics $P(\cdot|\cdot, a = x) = \mathcal{V}(x)$.
- Similarly, we can recover **pool-based active learning** (1), where the agent queries for labels for an entire pool of data points $\{x_1, x_2, \ldots\}$ where $x_i \in \mathcal{X}$ and receives a set of labels $\{y_1, y_2, \ldots\}$ where $y_i \in \mathcal{Y}$.
- Finally, we can also recover the final type of active learning: **stream-based active learning**. In this scenario, the agent has access to a stream of incoming inputs $x \in \mathcal{X}$ decides whether or not to ask for a label $y \in \mathcal{Y}$. So, $\mathcal{S} \in \mathcal{X} \times \mathcal{Y}$ and $\mathcal{A} \in [0, 1]$, where $a = 1$ indicates that we will receive a label $s = (x, y)$ and when $a = 0$, we will receive no label $s = (x, \emptyset)$.

These variants of active learning have been studied for tasks in computer vision and natural language processing, including topic classification (2, 3), object recognition (4), digit classification (5), and named entity recognition (6). Recently, methods have even proposed extending active learning methods that learn to generate questions: $\mathcal{A}$ is a set of possible natural language questions (7) and $\mathcal{V}$ is a visual question answering model. However, in all these methods, $\mathcal{R}_{\text{knowledge}}$ is the primary source of innovation, with methods that estimate $\mathcal{V}$'s least confidence measurements (8–10), epistemic uncertainty (11–13), Bayesian uncertainty (14, 15), disagreement (5, 16), and core-set selection (17). In all active learning methods introduced so far, α is set to 0, encouraging interactions that maximize the acquisition function $\mathcal{R}_{\text{knowledge}}$ and making the assumption that an oracle will provide new information for any interaction, therefore, ignoring $\mathcal{R}_{\text{interaction}}$. In contrast, by extending the framework as a reinforcement learning problem that characterizes social interactions within the reward $\mathcal{R}_{\text{interaction}}$, we hope that this will lead to a generalization of active learning where interactions involve real humans.

2. Protocols for interactive AI systems

Informed consent procedures. Our research was approved (protocol #50287) by Stanford University's Institutional Review Board (IRB) through an expedited non-medical review. Our IRB approves data collection from two online population pools: one from workers on Amazon Mechanical Turk and another from users worldwide on a social network.

We poll images from a social network, generate questions about concepts in the image and ask social network users by posting the question on their posted image. The questions are programmatically generated and vetted by Amazon Mechanical Turk workers as not being problematic or offensive. Only questions that are approved by workers are posted online to users.

Mechanical Turk workers are fully informed about the purpose of the study. They are told that we plan to generate questions would fit the social norms within the community and would be likely to receive an answer from an online social network user. Since our questions are automatically generated, workers are asked to identify questions that might be construed as offensive or rude to ask. They are informed that all questions that are vetted will be posted on social media. They are shown the image associated with the question but are not provided with links to the social network post or the poster's account.

Social network users are informed that we are asking a question about their image. All questions are preceded by the following introduction: "We are a computer science research project." The social network profile used to post the question also has the same message printed as its biography. Regardless of whether users respond, we debrief them of their participation by sending them a direct message on the social network after 48 hours of posting the question. We provide them with an email address in case they have further questions or reservations: "Thank you for responding to our question. Your answers will be used to improve an AI agent's ability to recognize concepts in images. Your original image and answer will not be released publicly. If you wish that we do not use your response or have questions about the study, please email us at <EMAIL_ADDRESS> or reply to this message."

Data privacy. We collect worker IDs from Mechanical Turk workers (which are anonymized). We also collect usernames for social media participants, which are publicly available (however usernames, personal information, etc. will not be used for any experiments or stored). Data is transferred using secured folders on Stanford University's AFS file system. Since our primary contribution is a framework and a proof-of-concept prototype, the data we collect will not be shared publicly. Participants are only be contacted by us if their posts are publicly accessible. We only collect publicly available data.

3. Designing a socially situated agent for social media

We argue that active learning efforts have myopically focused on only what the model requires, rather than what people want: what people are interested to label or identify, and the kinds of requests they are likely to respond to (18). Our socially situated AI framework outlines the various elements that need to be designed to deploy an agent in a social environment. The framework introduces socially situated learning as an iterative reinforcement learning problem with new rewards.

In this section, we describe the various components that were involved in deploying an agent on social media to improve its visual intelligence. We start by choosing the interaction modality and environment. Next, we design the inputs $\mathcal{X}$ and outputs $\mathcal{Y}$ for the computer vision model $\mathcal{V}$. Then, we formulate the state of the environment $\mathcal{S}$. Next, we draw on recent advances in natural language process and machine learning to design the agent's policy π, enabling it to explore the combinatorially vast space of interactions. Finally, we explain the parsing model, which extracts new concepts from user responses.

Application, environment, and mode of interaction. Modern computer vision systems rely on mountains of labeled training data, but generating labeled datasets remains challenging. In computer vision, for example, the ImageNet dataset (19) required fourteen million labels of basic human knowledge such as whether an image contains a chair. Unfortunately, this knowledge is both so simple that it is extremely tedious for humans to label, and also so tacit that it is often absent from the image's metadata. Although many volunteer labeling efforts have been deployed to incentivize labeling vision data (20–22), these methods have seen limited success. This combination of tedious and tacit leaves images online missing useful metadata describing its contents, and is why we chose it as the application domain to study socially situated AI.

Social networks are a popular platform for photo storage and sharing. Popular photo sharing platforms estimate that over $46,740$ pictures are uploaded every single minute with billions of active users. Given the large number of interactions centered around visual data on social networks, we chose it as our environment $\mathcal{E}$.

Since most information is exchanged using the comment section on image posts, we chose natural language as our interaction modality $\mathcal{A}$. To limit the scope of our experiments, we chose to design interactions by the agent to be a single question. Although one could extend our experiments to dialogues with multiple question-answering turns, we restricted our exploration to compare directly to active learning and existing data collection methods, where each piece of data is independently collected.

The computer vision model to optimize. Since we were using images with question-answers to collect data, we chose visual question answering as the computer vision model $\mathcal{V}$. It expects inputs $(i_t, q_t) \in \mathcal{X}$ to be an image and corresponding natural language question (23). It expects the output to be a natural language answer $ans_t \in \mathcal{Y}$. We use the popular stacked attention architecture for $\mathcal{V}$, though other architectures can be used if desired. The original architecture expects an image and a question as input and classifies an answer amongst a fixed set of 1000 answers ($\hat{a} = m(i, q)$). We modified the output of the original stacked attention model to generate an answer in natural language instead of just classifying within a fixed set of predefined categories. This allows our model to generate answers containing new concepts as it discovers them. Answer generation uses using a 2-layer LSTM with pretrained word embeddings from GloVe (24). So, as we learn about more concepts, we can use the pretrained GloVe embeddings to learn to answer questions about new concepts.

The knowledge reward. To ensure that our agent asks questions to learn new visual concepts, we design the knowledge reward. Drawing on active learning's uncertainty acquisition functions, we choose the knowledge reward to be $\mathcal{V}$'s uncertainty in answering a question, $\mathcal{R}_{\text{knowledge}} : (i, q) \to [0, 1]$. Intuitively, this encourages the agent to generate questions that $\mathcal{V}$ doesn't currently know how to answer. Typically, in the active learning literature, uncertainty is measured using entropy over all possible answers (8, 9). However, since we have an open vocabulary set of answers, measuring entropy is intractable because the normalization term requires measuring the probability of generating every possible answer. Instead, we approximate entropy by using beam search to generate the top 60 answers and measure entropy using these top 60 answers. We find that 60 is an empirically sufficient number of candidate answers to consider as the probability mass is concentrated within these top answers. We further normalize our approximate entropy measure to lie in the range $[0, 1]$. Our work is agnostic to any specific measurement of uncertainty and future work can explore other measurements.

The knowledge reward tracks the constantly shifting space of informative interactions as $\mathcal{V}$ learns. As our agent interactions with people, $\mathcal{V}$ uses the answers extracted from people's responses as training data. This re-training process updates which questions can now be answered. So, using $\mathcal{V}$'s uncertainty as a reward updates our agent to generate questions that result in informative responses.

The interaction reward. As the agent interacts with people online, it updates its policy to generate more socially acceptable interactions, which results in more responses. This goal is modeled using the *interaction reward*, $S : (i, q) \to [0, 1]$, to approximate which interactions result in a response containing the answer. Responses that contain an answer are used a positive (+1), and all other responses or lack thereof are used as negative (0) examples to train the function. The image and question are encoded into agent's policy $\pi_\theta(\cdot)$ and a two-layer LSTM question encoder $enc_\psi(\cdot)$; their representations are concatenated and used to regress onto a score using a learned linear transformation. The function is trained using a standard the mean squared error loss.

Language reward for the baseline agent. Since the baseline agent doesn't restrict its action space to the interaction representation, it can generate interactions using any sequence of words stitched together. Initially, the rewards learned are noisy, guiding the baseline to generate grammatically incorrect or incoherent questions. Existing state of the art dialogue generation agents have used a language modeling reward to encourage grammatically coherent generation (25, 26). We use the same reward to showcase that even with such a reward, there are no guarantees that the agent will be capable of quickly recovering useful questions when learning is restricted to a few thousand human interactions. We pre-train an LSTM language generation model on the questions about visual data (27, 28). The reward for a question is calculated as the inverse of the language model's perplexity. Intuitively, this reward encourages the generation of questions that grammatically resemble those found in available datasets. Our experiments indicate that when this reward's relative weight is set too high, the agent ignores the other rewards and doesn't deviate from its initialized behavior; similarly, when it is set too low, the agent quickly degenerates to producing nonsensical questions.

State of the environment. The formalization describes each environment state as containing $s_t = (i_t, ans_t)$, where i_t is a new image uploaded to the social network and ans_t is the human answer to the agent's previous question q_{t-1}. However, practically, we batch interactions to speed up training and average out the effects of any specific noisy interaction. So, practically, each state contains N' new images and N responses from the questions generated in the previous state: $s_t = (\{i_{t1}, \ldots, i_{tN'}\}, \{ans_{t1}, \ldots, ans_{tN}\})$. N' is $> N$ since we generate more questions than we receive responses. We filter new images $\{i_{t1}, \ldots, i_{tN'}\}$ to avoid asking questions about images that contain memes, cartoon, or ads (Section 6).

$\{ans_{t1}, \ldots, ans_{tN}\}$ is parsed (Section 7) to extract training data for $\mathcal{R}_{\text{interaction}}$ and $\mathcal{V}$. The updated rewards are then used to train the agent's policy. We set $N = 10K$, i.e. the rewards and policies are updated every 10,000 informative responses received.

Searching the space of possible interactions. The space of possible language interactions is combinatorially vast—the agent must learn to select the optimal set of tokens (words) to stitch together to form the optimal question. To decouple the agent's need to concurrently learn *what* interactions to initiate with *how* to generate those interactions, we utilize recent advances from machine learning. Specifically, we learn an lower dimensional interaction representation where questions about visual contents are likely to lie (see Figure S1). We use the interaction representation as a surrogate action space, which reduces the space of all possible interactions to a tractable search space. An agent's policy maps images encountered on social media to the interaction representation, $z \sim \pi_\theta(i)$. A decoder projects from the interaction representation to produce natural language questions $q \sim dec_\phi(z)$. Once the policy is initialized, the agent is deployed on social media to learn from social interactions with people.

Learning the interaction representation. Given the readily available Computer Vision datasets containing pairs of images and associated questions (27, 28), (i, q), the default approach to learning the interaction representation is to train a variational image-to-question generation model (29). This optimization maximizes the evidence lower bound (ELBO) or equivalently minimizes the following loss:

$$Loss(\theta, \phi) = -\mathop{\mathbb{E}}_{z\sim\pi_\theta(i)}[dec_\phi(q|z)] + D_{KL}[\pi_\theta(z|i)||p(z)] \quad [1]$$

where $\pi_\theta(\cdot)$ is the policy parameterized by θ and expects image i and generates an interaction representation z. Similarly, $dec_\phi(q|z)$ is the decoder parameterized by parameters ϕ and maps z to produce the question q. $p(z)$ is a uniform prior distribution. The first term maximizes the maximum likelihood estimation, or minimizes the reconstruction loss, of generating the associated question for a given image. The second term minimizes the Kullback-Leibler (KL) divergence of the categorical latent variables z with $p(z)$. Intuitively, minimizing KL-divergence with a uniform distribution is the same as maximizing the entropy of the predictions between the latent categories, encouraging the model to pick different categories for different questions.

This objective, as with many variational objectives, suffers from posterior collapse (30). Posterior collapse over z causes the decoder to produce near-deterministic outputs with little interesting variation since the latent categories are uninformative and ignored. Consequently, the decoder generates safe, overly general questions instead of learning the overall variance of all possible questions. Intuitively, this problem occurs because an image-to-question translation is a one-to-many mapping, i.e. a single image can create many questions. The model essentially learns to ignore all but one question. For example, it learns that asking "what color is the sky?" is a valid question for many images and resorts to asking that general question instead of focusing on other parts of the image.

To overcome posterior collapse in the interaction representation, we add a variational autoencoder objective that enforces that the interaction representation encodes and the decoder decodes a wide variety of questions for a given image. This new objective requires a new neural network module, $enc_\psi(\cdot)$, which learns to encode questions into z and is parameterized by ψ. This new question encoder is utilized to train the policy, the decoder.

Our new optimization loss is defined as:

$$Loss(\theta, \phi, \psi) = D_{KL}[enc_\psi(z|q)||p(z)] - \mathop{\mathbb{E}}_{z\sim enc_\psi(q)}[dec_\phi(q|z)] + D_{KL}[\pi_\theta(z|i)||enc_\psi(z|q)] \quad [2]$$

This optimization, specifically, the first and second terms, produces a one-to-one mapping from the input question to the output question by conditioning on a z, relieving the collapse and allowing the representation to learn to encode a wide variety of questions. The question encoder, $enc_\psi(\cdot)$, is used to train the question decoder instead of using the policy $\pi_\theta(\cdot)$. The third term uses the question encoder to train the policy, $\pi_\theta(\cdot)$. This term holds the weights of the question encoder constant and trains the policy to match the question encoder's outputs with a KL-divergence loss. This objective allows us to use the interaction representation as a surrogate action space that is lower dimensional than the complete space of possible interactions but is still expressive enough to represent a host of possible interactions.

We train $enc_\psi(\cdot)$ and $dec_\phi(\cdot)$ first and then finetune all three modules together. After which, we no longer need $enc_\psi(\cdot)$ as $\pi_\theta(\cdot)$ has already learned to pick image-relevant questions — and only use $\pi_\theta(\cdot)$ and $dec_\phi(\cdot)$ to generate questions from images.

When deployed on social media, we hold the decoder's weights constant and only update the policy to encode better latent interaction representations. Since we are utilizing the representation as a surrogate action space for reinforcement learning, we would ideally design the space to be large enough to represent the large variation of human-human interactions and small enough to learn without requiring hundreds of millions of interactions.

An obvious approach to try is designing the representation as a continuous d-dimensional space, constrained to lie within a multivariate Gaussian (30). However, we found it difficult to prevent the reinforcement learning updates from producing previously unseen continuous interaction representations. During training, the decoder sees values of z sampled from $enc_\psi(z|q)$ but never sees values sampled from $\pi_\theta(z|i)$, which is used during the deployment. In practice, we found that $\pi_\theta(z|i)$ changes sufficiently between reinforcement learning updates and leads to the generation of nonsensical questions. To overcome this challenge, we draw on recent work in dialogue systems, which suggest that discrete latent spaces (31) lead to more diverse language decoding (32) and are more consistent (33).

Therefore, we design the representation as pseudo-discrete using the Gumbel-Softmax relaxation (34). Specifically, the interaction representation is designed as m dimensions, each of which is a k-way classification. The interaction policy produces

m classifications, which are then embedded and utilized by the decoder to generate a question. This discretization leads to an action space of $m \times k$ while being able to represent k^m questions. We find that by limiting the space to a finite discrete space, we reduce the likelihood of generating out of distribution questions.

The decoder that generates question from the interaction representation. The decoder maps the m latent k-way categorizations in the interaction representation into a natural language question. We choose $m = 10$ and $k = 50$. First, it embeds the m latent variables, denoted as $z_j \forall j \in [1, m]$, into an embedding space: $e_j = E_j(z_j)$, where E_j is the embedding function for the jth latent variable. Since we have m latent embeddings $e_j \forall j \in [1, m]$, we need to decide how to utilize them in the decoding process.

Prior work has traditionally used a simple Long Short Term Memory (LSTM) network (35) to generate sequences, such as questions (29, 36). These approaches usually concatenate the latent embeddings into a single representation that can be used as the initial hidden representation for the LSTM. Today it is more popular to use an attention variant of the LSTM decoder such that at every time step, it uses its current hidden state to attend over the all the latent variables (37). This attention mechanism can be summarized by the following equations:

$$\alpha_{jt} = softmax(h_t^T W_a e_j) \;\; \forall j \in [1, m], \qquad c_t = \sum_{j=1}^{m} \alpha_{jt} e_j, \qquad \hat{h}_t = tanh(W_b \begin{bmatrix} c_t \\ h_t \end{bmatrix}) \qquad [3]$$

where h_t is the hidden representation generated by the LSTM at time step t, and W_a and W_b are linear layers with learned weights. α_{jt} is the attention weight over on the jth latent variable at time step t. $\hat{h}_t$ is the hidden representation sent to the next LSTM cell to generate the next word. Intuitively, this attention mechanism can be thought of as allowing the LSTM more capability by asking it to learn to focus on different latent variables at every time step instead of memorizing all the variables at the beginning and never being able to reference them mid-generation. We initialize the first hidden representation for the LSTM as $h_0 = \sum_{m=1}^{M} e_m$, which is often called a summation attention (33).

Our experiments are agnostic to any particular decoder model and recent successes in Transformer based sequence models might be a worthy exploration for future work (38).

Parsing responses from people. We need a response model that parses people's free form responses to our questions on social media. Our agent continuously polls to check if any of posted questions received a response. It continuously monitors posts for up to 48 hours. The likelihood of receiving a response after 48 hours drops significantly, so we categorize such posts as a negative interactions.

For the posts that do receive responses, those responses can include additional information beyond just the answer to the question (see Section 7). All responses are, therefore, parsed using a response model that extracts the answer from the freeform text response. The response model produces three outputs: (1) a binary flag indicating whether the the question was answered or whether the person was confused about the question, and (2) the start, and the (3) end indices of the response that contains the answer: $b, start, end = R(response)$ where $b = 1$ indicates that the person answered the question and $a = response[start : end]$ represents the extracted answer.

Our response model uses the Bidirectional Encoder Representations for Transformers (BERT-small) (39) model's pretrained contextual embeddings and fine-tunes them for our task using a dataset of 50,628 responses we collected from social media interactions (see Section 7). We tokenize each response and feed it to BERT, which outputs a representation per token. We pass these representations through a single fully connected layer that accumulates the final representations and attends over all the tokens to output a distribution over start and end spans. The accumulated representation also produces a single score $b \in [0, 1]$ with another linear layer and sigmoid activation. Since BERT performs subword tokenization, we align predicted start and end indices to their corresponding word tokens. While the response model is not a core contribution of this work, having a performant response model is crucial to the functioning of our agent (See Section 7 for more detailed analysis of the response model training and evaluation).

If the response model generates $b > 0.5$, then we infer that it has identified a response that contains an answer. With the answer, we generate a new training data point for the recognition model (i, q, a), consisting of the image, our agent's generated question, and the response model's extracted answer. This training data is used to update the recognition model. Each interaction generated, (i, q), is also sent to the social reward function, where it serves as a positive example if $b > 0.5$ or as a negative example if $b < 0.5$ or if the person never responds.

Updating the policy using interactions with people. With all our components in place, our agent can utilize its policy to generate interactions and learn from people's responses. The interaction policy generates a question, q given an image i:

$$z \sim \pi_\theta(i) \quad q \sim dec_\phi(z). \qquad [4]$$

The question is posted as a comment on the post associated with the image i.

Once the agent receives responses from N =10,000 people, the responses are parsed by the response model and used to generate training data $\{\{(i_0, q_0, ans_0), \ldots, (i_{N-1}, q_{N-1}, ans_{N-1})\}$ for $\mathcal{V}$. We batch the data into training batches of N to average out the gradients in a training step to avoid significant changes caused by a single noisy interaction. The reward functions are re-trained with the new data. Finally, the reward functions are used to train the interaction policy using proximal policy gradients (40) to maximize:

$$\theta^* = \arg\max_{\theta} \mathbb{E}_{z \sim \pi_\theta(i), q \sim dec_\phi(z)} [\sum_{n=0}^{N} \mathcal{R}(i_n, q_n)] \qquad [5]$$

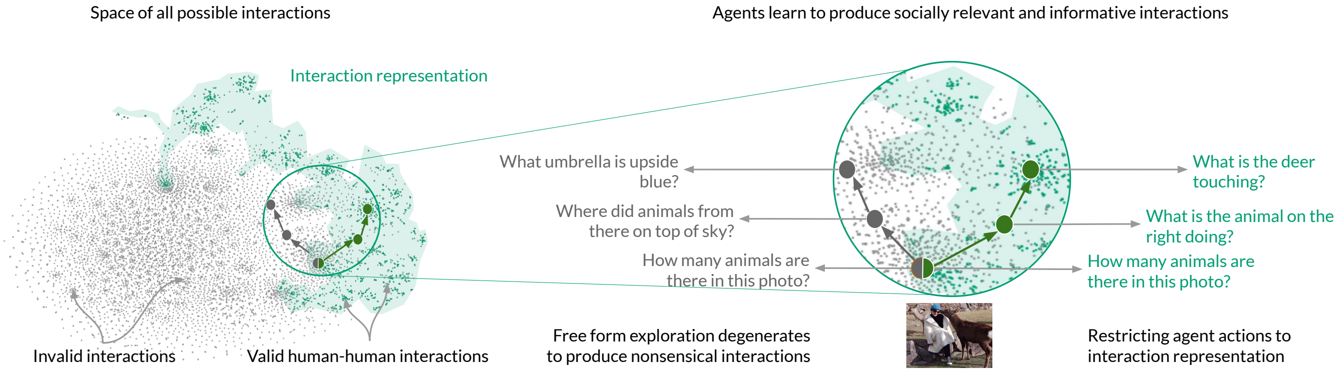

Fig. S1. The space of possible interactions is combinatorially vast and intractable for standard reinforcement learning agents to navigate, limiting their ability to learn from interactions with others. We use recent advances in machine learning to identify a lower dimensional *interaction representation* of real human-human interactions and uses it as a tractable surrogate action space. We visualize a t-SNE projection of the representation space of natural language questions about images. Green dots represent valid questions while gray dots represent nonsensical interactions. A standard sequence-to-sequence approach (visualized using the gray path) generates increasingly meaningless questions while restricting the agent's actions to the interaction space produces increasingly refined questions.

where

$$\mathcal{R}(i, q) = \alpha \mathcal{R}_{\text{interaction}}(i, q) + (1 - \alpha)\mathcal{R}_{\text{knowledge}}(i, q), \quad [6]$$
$$0 \leq \alpha \leq 1. \quad [7]$$

4. Related work comparison

Our work draws inspiration from a number of human-in-the-loop learning agents. We explore the various machine learning paradigms that influenced our design decisions, including question generation, active learning, lifelong learning, and reinforcement learning. Next, we place our work in context with conversational agents, interactive machine learning systems, and interactions through language.

Developmental robotics and reinforcement learning with humans. Reinforcement learning has gained popularity by achieving high performance in Atari games through deep q-learning and policy gradient methods (41, 42). Most reinforcement learning research makes two assumptions: (1) simulated environments with (2) a small action space. Work has begun to question the first assumption, engaging with human teachers (43). This line of work finds that people prefer to provide guidance instead of rewards (44–46) and provide more positive rewards than negative (47). Agents can minimize the amount of feedback required for training by modeling when they are uncertain about an action (48) or by visibly slowing down their action to indicate a need for human assistance (49). With these design decisions in mind, the Cobot and TAMER developmental robotics frameworks (50–52) build agents that use rewards from trained human participants. Our work generalizes these approaches by learning how to interact with people in social environments without needing to train people to interact with our agent. Our framework infers rewards implicitly from human interactions as opposed to these methods, which teach people to provide explicit rewards. Additionally, prior work has still to address the second assumption, the small action space. In particular, our work is the first to explore a language-based reinforcement learning agent with rewards attained from direct human conversation.

Question generation. In the field of Natural Language Processing, a few methods have attempted to automatically generate questions from knowledge bases, using rule based (53) or deep learning based approaches (54). In Computer Vision, a few recent projects have explored the task of visual question generation (36, 55). These projects have also either followed an algorithmic rule-based(53, 56) or learning-based(57, 58) approach. Newer papers have treated the generation process as a variational process (29, 36) or placed it within an active learning (7) or reinforcement learning framework (55). The closest related work to ours frames question generation in the context of synthetic images and simulated oracles who respond to all queries (7). Our work draws inspiration from these previous methods by using question as a modality for continuous learning. It also draws on recent information maximization work to initialize the question generator within a variational autoencoding framework.(29) We introduce a latent discretization of the question topic using the latent interaction respresentation, allowing our agent to tractably explore and unearth useful social interactions.

Active learning. Active learning is an machine learning paradigm where a model iteratively maximizes its performance while minimizing the number of annotations (1). Typical strategies for choosing which data points to label involve formulating an approximation of model uncertainty such as entropy, least confidence measurements, or the expected impact on the model (8–10). Active learning has successfully been deployed with crowd workers to improve the state of the art on large scale tasks (13, 59, 60). Unfortunately, recent work has concluded that users don't want to serve as simple oracles by repeatedly providing labels, breaking a fundamental assumption in active learning (61–63).

Our framework is a reaction to this observation: active learning doesn't take into account what others are willing to teach in realistic social environments (see Section 12). We seek to learn which requests are acceptable within a given social context—concepts that people are willing to teach us should be as important of the concepts we want to learn. Furthermore, we study data acquisition through natural questions, generalizing most existing active learning methods, which focuses primarily on obtaining classification decisions (8, 9).

Lifelong and never-ending learning. We draw inspiration from prior work on lifelong learning and never-ending learning. In lifelong learning, models are trained continuously to accumulate knowledge over time (64–66). In never-ending learning, the latest models are trained to scrape new information from the web and iteratively retrain (67–72). Unlike these approaches, we enable the collection of novel or tacit information that is not already available online. No amount of re-reading existing web pages will enable these approaches to obtain information that is simply not present or well-structured enough to be extracted; but, by asking questions, our prototype agent can quickly bootstraps new knowledge. Unlike never-ending learning (70), our framework is task-focused: it directly aims to improve performance on an underlying model's learning goal.

Curiosity and intrinsic motivation Our work is also related to recent work on curiosity-based exploration strategies in reinforcement learning (73, 74). Existing methods have explored strategies that guide AI agents to discover novel states (75–77) or surprising behaviors (78–81). Recent work has also shown that socially situated robots can learn to choose amongst a small set of predefined actions when interacting with real people (82). Our work draws on these ideas and uses uncertainty to design the knowledge reward to decide which states to explore and which to avoid. Unlike previous work, our prototype explores a knowledge-based motivation in a large space of states and actions.

Interactive machine learning. Over the last decade, there has been a shift towards considering the role of end users in machine learning systems (83). Crayons was the first such system, demonstrating that end users can quickly author ad-hoc classifiers through demonstration (84). Follow-on work generalized this technique to other domains such as image search and more directly integrated an understanding of the successes and vagaries of human labeling behavior (85). At a professional level, developing machine learning systems is an iterative process that can be difficult to understand or evaluate (86). Generalizing common machine learning workflows can help developers quickly transition back and forth between implementation and analysis (87). Likewise, iterative reflection interfaces allow annotators to update their decisions and improve the labeling quality (88, 89). Machine teaching and play-along learning explore the role of a human teacher in providing guidance to an agent to improve its performance (90, 91). Today, developing machine learning systems is a complex interactive process, where users manage, version, customize, and reuse both their data and their model components (92). Given this complexity, recent work has also proposed a set of design guidelines to help developers navigate the space human-AI interactions (93). Our framework builds on this work by generalizing to contexts where the user's main goal is not to train a model but to simply engage in human-human interactions; by modeling people's interests, our agent shows that it is possible to extract learning signals from social interactions.

Interaction through language. The use of natural language as a medium for interaction has spurred many systems (94–96). Natural language enables commands in complicated design tools such as GIMP (97), clothing searches in fashion databases (98), visualization authoring (99), language teaching (100), and image editing (101). Users often express their goals through natural language, then author or integrate computer-readable code as a result (100, 102). We extend this work: rather than using language as an input command language, we utilize it as an interaction modality for intelligent agents on social media, without any restrictions on vocabulary or grammar.

Conversational agents. For over half a century, researchers have been studying how to program computers to participate in open-ended conversations (103). Though initial programs were hand-crafted to handle simulated environments (104), recent agents can guide a user through a real world data science workflow (105). Today's deep learning dialogue agents (26) are capable of engaging in long conversations with users and utilize language modeling rewards when learning from human interactions to prevent the agent from veering off its initialization (25). Conversational assistants have also moved from research into products, such as Apple's Siri, Amazon's Alexa, and Google's Assistant. These agents, however, are currently limited to specific speech commands that have been coded for pre-determined domains (106–112). As a result, substantial effort has been placed on teaching people how to talk to these assistants. Noticing this limitation, more robust crowd-powered conversational systems have been created by hiring professionals, as in the case of Facebook M (113), or crowd workers (96, 114). Although impractical for deployment because of their high latency and cost, crowd-machine hybrid systems have emerged as a solution that combines the robustness of crowds with the speed of automated agents (95, 115–117). Unlike the past research on conversational agents where humans have a goal and invoke a passive conversational agent, we take an alternative approach—our framework instantiates active agents that engage people in conversations to express its knowledge gaps and accomplish its own learning goal.

Asking questions on social media. Today, broadcasting questions on social media is a common occurrence, giving rise to specialized question-answering platforms like Quora and StackOverflow (118, 119). Numerous studies have noted that the phenomenon of *social search* is not limited to such specialized platforms but abundant in most networks, including Twitter, Instagram, and Facebook (120–122). These studies have been identifying and predicting what questions people post online (120), the quality of answers they receive (118, 123), user satisfaction (124, 125), learning to rank answers (126, 127), identifying motivations for answering (122, 128, 129), and modeling the user authority and level of expertise (130). While we do not directly study what kinds of questions are likely to succeed, we find that our agent's emergent behavior is consistent with strategies employed by people. Namely, our deployed agent learns to ask shorter questions, ground objects in its questions to establish social proof, ask fewer vague questions, prefer questions that can be easily answered.

5. Data workflow for social media deployment

In this section, we detail our agent's workflow — how it interacts with people online and uses the responses to update itself (see Figure S2). To reiterate, once the interaction policy and the decoder are initialized, the agent is ready to interact with people on social media. Through each interaction, the agent learns to improve its ability to generate better questions while also improving its visual intelligence.

Our agent searches for new posts on social media and filters images to interact with. Images that pass through the filter are embedded into a representation space, from which the decode generates questions. Once initialized, the decoder's parameters are not updated, even as the agent interacts with people to collect new data. The generated question is posted back to the social media post as a comment. For example, it can post "Is that animal on the tree a fox?". If the question receives a response from the poster, the response is parsed using a pre-trained parsing model. Responses that contain an answer, e.g. "it's a red panda", are treated as a positive reward for the interaction reward and the answer is used as new training data for the recognition model. The recognition model's uncertainty is used as the knowledge reward. If the question does not receive a response or if the response doesn't narrow the space of possible answers, e.g. "it's not a fox", it is treated as a negative interaction reward.

The two rewards, the interaction and knowledge rewards, are updated after 10,000 answers are received. They in turn serve as reward functions to update the interaction policy. Once updated, the agent continues interacting with more people on social media to dynamically continue learning.

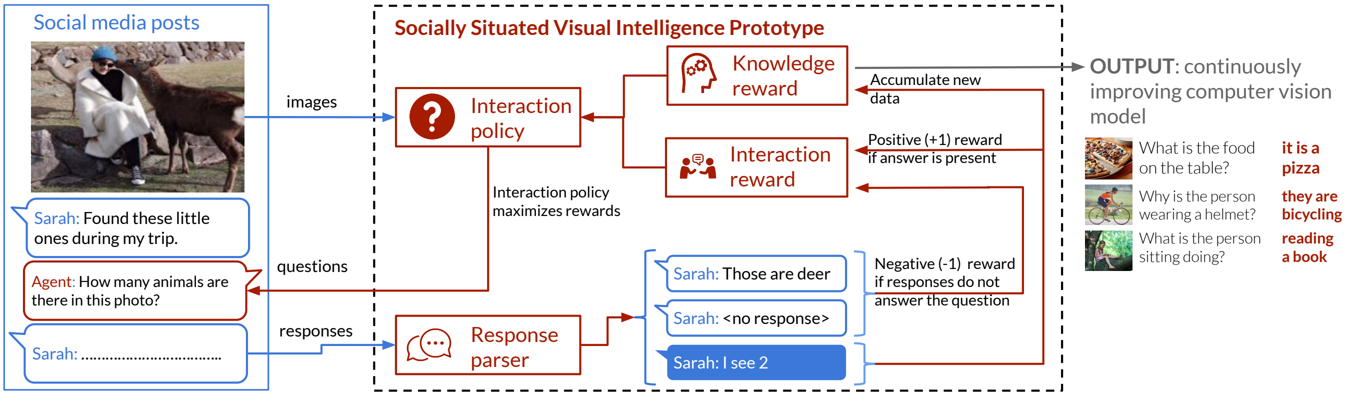

Fig. S2. Overall system diagram depicting the various components of our deployment. The agent filters through recently uploaded images to find concepts that it doesn't currently recognize and generates questions to ask people. These questions are posted as comments to social media posts. Each poster's response is parsed and used as either a positive or negative interaction reward, depending on whether the the response contains the answer to the question. The answers are also used as new data to re-train and continuously improve a computer vision model, which is used to calibrate the knowledge reward. After receiving a certain number of answers, the rewards are used to update the interaction policy. Overtime, the agent learns to generate questions that are more likely to receive responses while simultaneously updating the computer vision model.

6. Filtering images

Our agent only interacts with *new* social media images that have been posted because it is unusual to find interactions between users on older posts. We poll images that contain hashtags related to food, furniture, fashion, nature, sports, or animals (see Table S1). We only interact with image posts that have been posted for at least one hour, because we find that two thirds of social media images are deleted within the first few minutes of posting. We also check for duplicate images that we have already interacted with.

Images posted to social media can contain memes, cartoons, advertisements or other content that does not teach the AI model about the visual world. Therefore, all images are filtered by a trained classifier to remove cartoons, animations, videos, images containing any text, and inappropriate content. We train the classifier by curating a dataset of $100K$ real photos and $100K$ unwanted content. We train the filter by finetuning ResNet50 (131), pretrained on ImageNet (19) using a dataset of $100K$ images collected by polling hashtags that contain unwanted images. The dataset contains an even split between real images and other content. We evaluate the filter on a held out test set, which we manually annotate using online workers from Amazon Mechanical Turk. The filter achieves a precision of 97% and recall of 78%. Precision is the important metric in this evaluation as we want to minimize the amount of noise passed through our system.

During deployment, around 33% of images typically remain after the filter. These images are sent to the agent. The rest are discarded.

7. How well do we parse responses?

In this section, we analyze the responses received by our agent from users on social media over a deployment of 8 months. We detail how the parsing model was trained and compare its performance, against existing baselines, at classifying whether the user answers the question, and at extracting answers from the responses.

Dataset. Prior to our deployment, we manually annotated 50,628 responses from social media users, gathered over 2 months. The questions were generated by the initial agent and verified by paid annotators hired from Amazon Mechanical Turk. Workers were asked to verify whether each question was answerable, given only the image, i.e. "would you be able to answer this question about this image?". Questions that passed the verification were posted to social media. The responses were annotated using additional paid annotators, who assigned binary classification labels indicating whether the response contained an answer, and indicated the answer span in the response. Crowd workers who verified each response were not necessarily the same ones who annotated the answer spans for a particular response. We randomly sampled our dataset into 80%/10%/10% train/val/test splits to train the response model.

Response statistics. Our agent received a wide range of responses, ranging from short 1 word responses to as long as 100 words with a mean of 35.9 characters and 6.8 words. In contrast, the average word length of answers in the VQA 2.0 dataset(28) is 4.5 words. Out of the 2 million words in the responses that we annotated from 50,628 responses, there were a total of 54,877 unique words. In contrast, the VQA dataset has 22,234 unique words in 10 million total answers manually generated by paid crowd workers: half the unique words in five times the data. We find that when users reply, 67% answer the question.

When users receive an irrelevant question, they usually respond asking for clarifications, suggesting that they are willing to engage in future exchanges. For example, when asked "where are the people ?" to a picture with no people, the user responded "only food on this post aha !". In another example, when asked "what is the person holding?" to a picture with multiple people, the user responded "which person?".

Users' affective response to our agent vary: some users treat our agent with matter of fact answers, others use it as an opportunity to get a new follow and follow our agent back. Some tell us long detailed stories about their images (see Figure S3).

Metrics. We evaluate performance on the binary classification of whether an answer exists by computing precision, recall and F1. Performance on the answer span localization is evaluated with F1 and exact match scores, following the standard established by the Natural Language Processing community (132), where the task requires models to extract answers from passages.

Baselines. We compare our BERT-based model against two baseline models: (1) a bag of words and (2) GloVe embeddings (24). The bag of words model performs a naïve tokenization of words in questions and responses, and generates a fixed vocabulary from the 10,000 most frequent words appearing in the training set. All questions and responses are then encoded as bag-of-words frequency vectors of size 10,000. The question vector and response vector are individually passed through separate fully-connected layers and ReLU non-linearities, before being concatenated and passed through a final fully-connected layer. The GloVe model uses pre-trained GloVe embeddings instead of bag-of-words, encoding the question and response using an LSTM (35) before concatenating them and passing through a linear layer.

Performance. Across all of our metrics reported in Table S2, we find that the BERT response model performs much better than the baselines we considered, achieving improvements of 17 F1 points over GloVe on the answer-exists task, which represents a 32% relative improvement. Jointly training the response model on both tasks performs slightly better, by 1 to 2 F1 points, than when the model is trained on each task individually. By performing the two tasks in tandem, the model is able to learn from its shared representations and perform better together. This performance gap is consistent with existing language processing tasks explored in the original BERT paper (39).

Error analysis. It is informative to consider the errors that the response model can make. Most of the errors incurred by the response model are a direct consequence of our agent generating questions that are irrelevant to the image. For example, consider this exchange: our agent asks "What is on top of the cake?" and the response is "chilli, that is not cake that's chicken".

Table S1. Categories of images polled on social media. We poll images using 30 different hashtags across 6 categories. These categories were chosen heuristically by exploring which of the top 500 hashtags contain mainly natural images and not memes or cartoons.

Category	Hashtags (#)
Food	eathealth, foodporn, eeeeeats, foodandwine, nomnomnom, tryitordiet, buzzfeast, forkyeah
Furniture	interiordesign, home, furniture, furnituredesign, livingroom, homesweethome, homeinterior
Fashion	dress, outfit, clothes, fashiongram, fashionista, fashionblog
Animals	dogsofinstagram, catsofinstagram, cats, pet, petsofinstagram
Sports	athletics, soccer_nation
Nature	nature, forest

Table S2. The BERT-based response model we use performs better than simple GloVe embeddings by $.17$ F1, a 32% relative improvement, at detecting whether the user has answered the question. Its F1 score at classifying the span of the answer at 0.74 indicates that our agent can gather useful data from the responses.

	Answer exists?			Answer Span Prediction	
Model	Precision	Recall	F1	Exact match	F1
Bag of Words	0.37	0.18	0.24	N/A	N/A
GloVe embeddings	0.62	0.46	0.53	N/A	N/A
BERT (individually trained)	0.71	0.66	0.68	0.57	0.74
BERT (jointly trained)	**0.73**	**0.67**	**0.70**	**0.60**	**0.77**

This causes the response model to output “chilli” as the correct answer span along with the incorrect prediction that the answer does not exist, likely because it misinterpreted “that is not a cake” to imply an irrelevant question. In another instance, our agent and the user do not reach a shared understanding: our agent asks “What is on the building”, and the user responds “it’s a kindergarten”. The user corrects our agent’s classification of the building by noting that it is a kindergarten, but doesn’t actually answer the question. The response model however, predicts incorrectly that something called “kindergarden” is on the building. Finally, occasionally the response model only selects half of the correct answer and ignores half the answer. For the question “What is on the table?” and the response “beet and carrot juice”, it predicts the answer span as only “juice”.

In summary, we receive a wide variety of responses from social media, and our response model is effective at extracting answers from the responses.

8. How are the questions vetted before posting online?

Human oversight is a necessary step when deploying AI systems that interact with humans. Human oversight, in the form of vetting (133, 134), or editing (95), is a common practice across numerous socio-technical systems; for example, the old Aardvark social search engine answered users’ queries by initially hiring their own employees to serve search results (134); similarly, today’s content moderation research enthusiastically advocates for the irreplaceable need for human oversight of AI predictions (133).

Similar to existing literature, we advocate that interactive AI systems should not operate completely autonomously; human oversight should always be present. Our deployed system employs human oversight by vetting questions before they are posted to users online. We hire workers from Amazon Mechanical Turk (AMT) for such oversight.

Workflow. Workers were chosen from Amazon Mechanical Turk; we chose workers who have completed at least 10,000 HITs and have a 97% approval rating. Workers are asked to analyze all questions generated by the AI agent before the questions are posted online. Workers are presented with the image, the post associated with the social network post, and the AI’s generated question. They are asked to identify questions that could be interpreted as offensive or socially inappropriate to ask. Identified questions are rejected and not posted to users online. Workers are asked not to reject questions for other reasons; for example, they are asked not to reject questions that make incorrect references to objects in the image or are that are irrelevant to the image, even if the question could not be answered from the image. We provided workers with 5 example questions we had identified as potentially offensive. We also provided 5 example questions that were irrelevant but not offensive.

Analysis. During our deployment, workers identified 2.4% of all AI questions generated as potentially offensive or rude. Through a qualitative analysis, we uncovered that workers rejected questions that inappropriately refer to posters’ age, clothing choices, personal items, living conditions, or food preferences. For instance, questions such as "are they wearing a bib?" assumes that the person in the image is a child; the training datasets used to initialize our agent’s question generator contains many images of children being fed at home while most images on the social network do not. Similarly, questions assuming that people were in "costume": "why are they in costume?" are rejected to avoid referring to someone’s outfit as a costume. Some questions in the original dataset referred to people’s undergarments; questions generated with such references are also rejected. Workers rejected questions that could be construed as insulting people’s food or living preferences: "Is that edible?" and "is this desk messy?".

We ensure that the rejected questions are incorporated into our evaluation metrics: questions rejected are counted as uninformative interactions. In other words, if our AI agent changes its behavior towards producing more rejected questions, it would decrease our response rate; this change would be reflected in our informative response rate metric. Therefore, the human vetting step decreases our informative response rate. If we had posted the rejected questions, some of those questions might have received responses and might have led to an increase in our recognition accuracy. However, as we advocated for earlier, we chose to trade off the possibility of learning something new in return for safer interactions facilitated by human oversight. We have added more information about the impact of this vetting step in the Supplementary materials section on human vetting.

If we had posted the rejected questions, some of those questions might have received responses and might have led to an increase in our recognition accuracy. However, as we advocated for earlier, we chose to trade off the possibility of learning something new in return for safer interactions facilitated by human oversight.

9. How are the questions edited by people different than those generated by agents?

Procedure. To contextualize our agent’s ability to engage people on social media and garner responses, we report the modifications people make to their questions to make them more likely to receive a response.. We hired Amazon Mechanical Turk (AMT) workers to write questions for social media images. Workers were paid an equivalent wage of \$12-\$15 an hour. We sourced all our images from social media in the same procedure as our other experiments. Workers were able to generate questions and invoke our recognition model to see if it can already answer the question. They were instructed to write questions about the contents of the image that the recognition model answers incorrectly but could be correctly answered by a person. They were also encouraged to rephrase the question to increase the likelihood of a response from the person who originally posted the social media image.

We did not set any maximum edit distance constraints for the annotators. The only constraint we imposed was to ensure that the original intent of the question was not altered. For example, a bad edit would change "Do you work here?" to "Is this where you work because I would love a setup like this?". Such an edit would change the original intention of the question (see Figure S5).

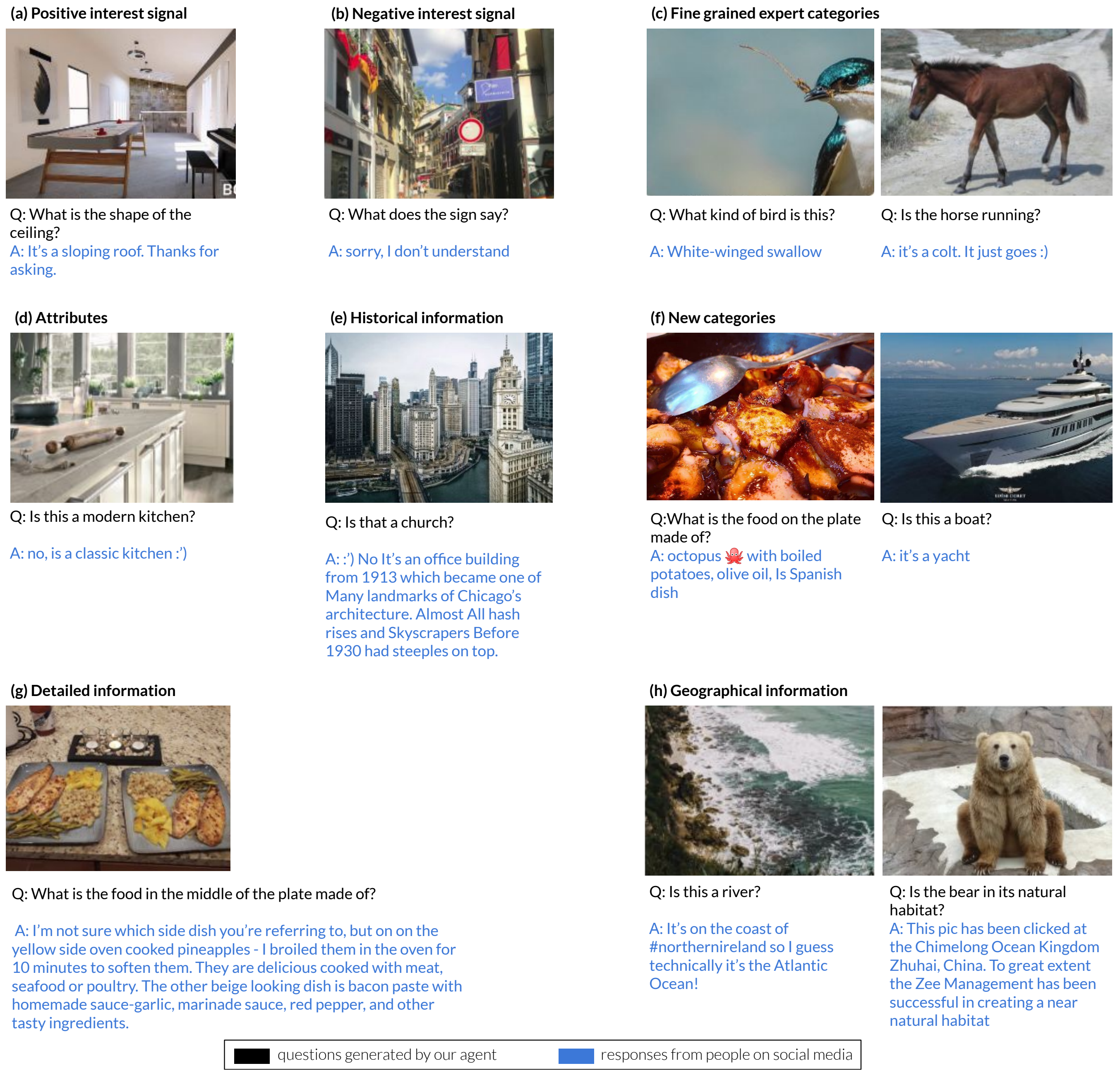

Fig. S3. A qualitative categorization of the variety of rich answers we receive. **(a, b)**: Some responses carry along additional signal when our questions result in positive or negative engagement. **(c)**: Many responses result in rare classifications of fine grained categories that are difficult to attain without expert knowledge. **(d, e)**: People also provide object attributes and even historical information outside the context of the image. **(f)**: Responses often contain new unseen categories. **(g)**: People answer our questions in many unspecified variations, making it difficult to parse long detailed annotations. **(h)**: We receive geo-locations of where a particular image was taken.

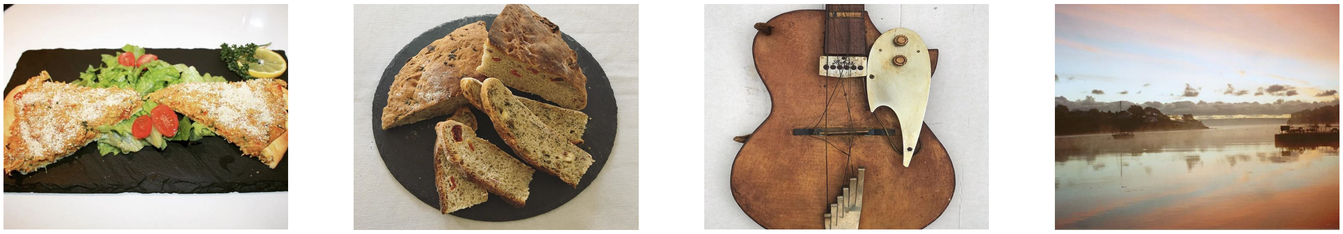

Q: that is very good looking, what is the name of the dish?
A: it is a caribbean dish named << crab pie >>, very tasty!

Q: What type of bread is this? It looks like a sourdough with something in it.
A: yes, there are sun dried tomatoes and beet greens in it.

Q: this type of art is called what, i have seen it before?
A: looks a bit steampunk I suppose, but created well before that term was thought of

Q: I love the colors in this, was it edited in any way or natural?
A: thank you so much, not edited at all, just nature doing its best work! 🌄😁

questions edited by hired workers — response from people on social media

Fig. S4. We visualize a sample of questions edited by online annotators when instructed to modify questions to increase the likelihood of a response. We tasked workers with writing questions that our recognition model could use to learn new visual concepts and also asked them to generate the questions in a manner most likely to receive a response from social media. Here, we show some examples that people edited.

Instructions

- We are trying to write an algorithm that can recognize everything that is in pictures.
- In this task, you will be shown a picture that someone uploaded to their social media account with a caption. You will be shown a question generated by the algorithm. The question is trying to learn about the contents of the picture
- Your goal is to edit the question so that the person who posted the social media picture is more likely to answer the question.
- In some cases, the algorithm might ask a question that might be unanswerable. Sometimes the question might even be irrelevant to the image. For example, sometimes the question might ask "what color is the bottle?" but the picture might not contain a "bottle". If you encounter such questions, DO NOT modify the original intenti of the question. Assume that the question is correct: let's assume that there is actually a bottle in the image. Edit the question to make it more likely for someone to respond to the question assuming that the question is correct.

Here are some example questions edits:

Example 1

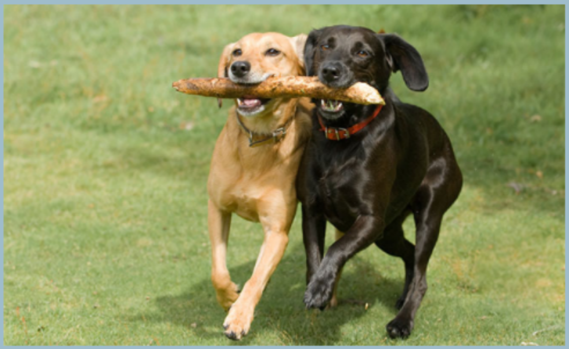

Here are some GOOD edits you can make:

- What are these cute dogs doing with the stick? can be edited to What are these cute dogs doing with the stick?
- What breed is the dog on the left? can be edited to Those are such cute dogs. What breed is the dog on the left?

Here are some BAD ways of editins a question because it changes the original intent of the question:

- Is that your dog? should not be changed to Is that your cat?
- Does your dog like to play a lot? should not be changed to Does your Does like to run a lot?

Example 2

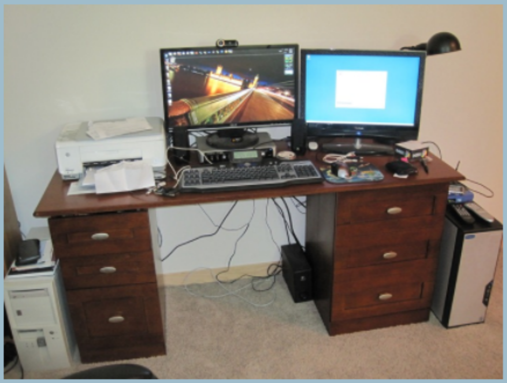

Here are some GOOD edits you can make:

- What is that thing above the computer screen? can be edited to That's a nice setup. What is that thing above the computer screen?
- What is the black box below the desk?What is the black box below the desk? I can't recognize that curious item.

Here are some BAD ways of editins a question because it changes the original intent of the question:

- Is it possible to build this yourself? should not be changed to Is that a new computer?
- Could you work here? should not be changed to Is this where you work because I would love a setup like this?
- Where was the photo taken? should not be changed to Is this your work space in your house?

Fig. S5. Instructions provided to workers for editing the questions generated by our agent.

Results. Examples of questions edited by workers are shown in Figure S4. We collected a total of 19,302 human edited questions and posted them to social media as comments, similar to our other experiments. According to a two proportion z-test, questions edited by people received a response rate of 37.00%, which was significantly higher than our agent at 33.32% ($z = 11.54, p < 0.001$). Workers' questions were also on average 11.58 ± 3.50 words; in contrast, our agent's edited questions are 6.81 ± 1.00 words, which is significantly shorter according to a paired t-test ($t(19596) = 229, p < 0.001$).

Out of the $10K$ vocabulary words available to our agent, it produced questions using only 5414 unique words. Meanwhile, crowd workers used 8414 unique words in their questions, of which 7472 words were not even part of the readily available data used to train our interaction representation. This result indicates that 88% of words used by workers weren't even present in standardized Computer Vision datasets like VQA v2.0 and Visual Genome v1.4, which was used to train our question decoder. This result highlights an imperative need for new large scale datasets that capture natural conversations between people around their visual content.

To characterize the gap between our agent's current abilities and human questions, we qualitatively analyzed a sample of the questions edited by paid crowd workers. First, people often complimented the picture before asking a question: "that is a very good looking dish, what is the name of the dish?". Second, they followed up questions with guesses: "what type of bread is it? It looks like sourdough with something on it". Third, they asked questions that would be impossible to answer about an image without having prior background knowledge "this type of art is called what, i have seen it before?". The current instantiation of our agent does not yet apply these strategies (122, 135), because its underlying dataset was not created with the goal of replicating social media conversations, and were intended to be as dry and factual. Our agent's rewards were configured such that they did not encourage the generation of meaningful or more human-like interactions; instead, they rewarded the initiation of interactions that elicit new information, regardless of how natural they appear.

10. Test set construction

The purpose of our experiments is to demonstrate the possibility of socially situated AI, i.e. agents that can learn how to interact with people in order to learn from those interactions. Unfortunately, our preliminary pilot experiments demonstrated that the categories of objects that people typically post pictures about on social media today focus on concepts that are very different from those found in popular computer vision datasets. Therefore, evaluating a model trained using social media data requires a new test set collected using social media. Therefore, to evaluate whether the data collected from human interactions are useful, we curated a test set.

In most computer vision tasks, it is common practice to collect training and test sets from the same data distribution (19, 136). Following tradition, we collect our test set by deploying the initial agent to ask questions on the social network. The responses elicited are sent to Amazon Mechanical Turk annotators to verify, i.e. make sure the questions are grammatically valid and answerable by looking at the image. The questions that were verified by annotators were posted on social media. The responses to these questions were sent to another set of annotators, who extracted the answers to the questions. Using this workflow, we collected a test dataset of $80,234K$ pairs of images, questions and answers. Since the initial agent's response rate was 22%, we curated this test set of $80,234K$ images with questions and answers by asking $367,382$ questions online for 2 months.

Like most data curation processes, we witnessed a long tail distribution of concepts annotated by human users. Mechanical turk annotators extracted $9,236$ unique answers from the responses. Of those, we curated a test set containing just the top 1000 most frequent answers. This test set contained 50,104 images with questions and answers. 418 of those categories are new categories unseen in existing test sets (Figured S6).

11. How well does the computer vision model perform?

Our aim is to demonstrate the possibility of learning through social interactions with people in social environments. As machines are deployed, a socially situated learning paradigm acknowledges that agents will eventually encounter unfamiliar scenarios and must learn new concepts. In comparison, machine learning models are trained using standard datasets and simulations and expected to work in real-world settings. In this section, we dive deeper into the importance of allowing agents to adapt and train through a socially situated process. We compare the vision model $\mathcal{V}$, which was trained using data collected by our socially situated agent versus a standard model trained using data from existing computer vision datasets (28).

Our experiments show that data from existing datasets result in models that show strong test set performance in their associated standard test set but perform worse from data collected in real world settings, such as social media. Similarly, we show that training data collected in order to adapt to an environment will result in better performance in that domain, even if the same improvements are not measured by standard test sets. These results, taken together, provide additional evidence to support enabling agents to socially adapt and improve their capabilities in the domains they are deployed in.

Test datasets. We measure recognition performance on two datasets: the original VQA 2.0 dataset(28) and the test set collected from social media. There is a difference in the distribution of images and concepts between the two datasets, as our agent sources images from social media while the VQA 2.0 dataset's images were curated from Flickr. We report the performance of our models on both datasets.

Metrics. To measure recognition performance, we report accuracy in picking the correct answer out of a list of possible answers. For both datasets, there are 1000 possible answers (just like the VQA 2.0 dataset, we only evaluate on the top 1000 answers found in our test dataset).

Results on social media test dataset. Models trained from examples collected from interactions with people lead to a 21% increase in accuracy on our social media test dataset when compared against models trained on traditional benchmark datasets.

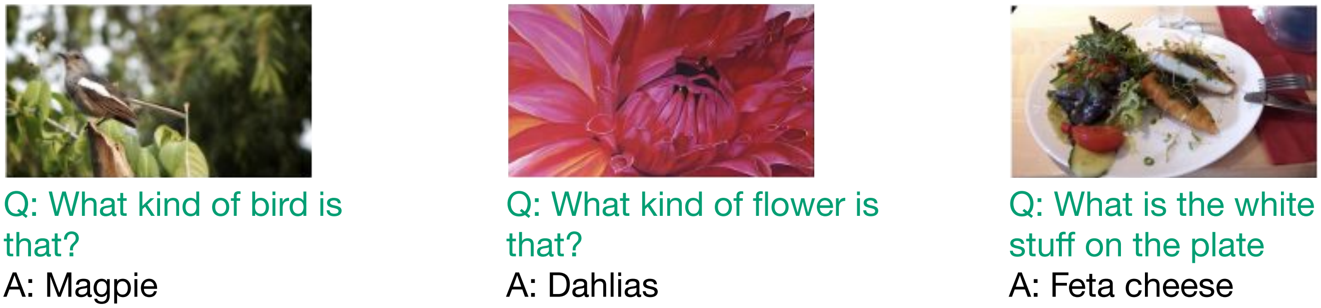

Fig. S6. Example new categories in the social media test set. Photos on social networks often reference animal and flower species and popular food items. Since these categories are not present in popular computer vision benchmark datasets, correctly identifying these categories are improbable. Only by learning to ask questions to people who potentially encountered these categories in the real world, will agents curate useful training data for a model to identify such categories.

Evaluating on the social media test dataset represents utility of our socially situated learning on a real-world domain of images found on social media. Figure S7(a) visualizes performances of the two models on our social media test dataset. Training on all $70K$ (100% of) training examples collected from interactions by our agent results in an accuracy of 39.44% while training on an equivalent number of labels in VQA 2.0 (15% of its volume) only achieves an accuracy of 17.45%. Additionally, we find that even when training on all $443K$ (100% of) labels in the VQA dataset, the model's performance reaches 28.55%, which is still 10.89% lower than what our method achieves with only a fraction of the data.

Results on VQA 2.0 dataset. Evaluating on VQA 2.0 dataset further demonstrate the mismatch between real-world and traditional benchmark datasets. Figure S7(b) visualizes performances of the two models evaluated on the VQA 2.0 dataset. This is a much harder task for the model trained on social media images since the concepts and image encountered on social media are very different than those in VQA 2.0. We should expect models trained using social media data to perform poorly when compared against models trained using training data from the same distribution. As expected, when trained with the entire $443K$ (100% of) labels in VQA 2.0, the model achieves an accuracy of 75.70%, while training from the examples collected from interactions scores 37.23%. However, our model (at 100%) does achieve parity with VQA when VQA is trained on a smaller set (15%), and its derivative is still positive. This experiment demonstrates the importance of deploying agents to learn from social interactions with people in whichever domain they are deployed in.

In summary, our agent manages to improve a vision model from its interactions with people on social media. We also find that the interactions leads to improvements in both traditional benchmarked datasets and shows better performance when the training examples are collected from interactions in the same distribution. Our experiments showcase the possibility of building agents to improve their performance by adapting to new domains and social situations by interacting with other people also present in those situations.

12. Is the interaction reward required?

In our experiments, we utilize two separate reward functions to help guide the agent towards achieving its two goals: (1) The interaction reward incentivizes the agent to initiate interactions that people want to respond to, and (2) the knowledge reward incentivizes the agent to ask questions its current recognition model is uncertain about. In this section, we dive deeper into how these two rewards interact using the ablation that trains using only the knowledge reward and ignores the interaction reward. This experiment measures the impact of the first goal on the second, i.e. if the agent didn't consider which interactions people are interested in engaging with, how will it impact its recognition ability?

Experimental setup. We launch both ablations of the agent on social media during the same 8 month deployment. One ablation only utilized the knowledge reward function while the second ablation, our main agent, utilizes both rewards. Each agent's rewards and interaction policy are updated after every 10,000 interactions.

Metrics. We measure progress towards the two goals using the same metrics as the rest of the paper: (1) informative response rate measures the percentage of interactions that are relevant to the image, answerable and receive an answer, and (2) accuracy measures the percentage of questions that the recognition model answers correctly in our social media test dataset.

Results. We find that when our agent only uses the knowledge reward and doesn't utilize the social interaction reward, its informative response rate drops over multiple interactions and its recognition accuracy increases at a slower rate. During the deployment, the agent that only uses the knowledge reward function initiated 274,893 interactions, out of which, 40,000 people responded with an answer. Its informative response rate dropped from the initial 22% to 12.3%. Its recognition accuracy improved from 18.13% to 31.4%. In comparison, our agent, using both rewards, initiated 236,106 interactions, out of which it receives responses from 70,000 people. Its informative response rate increases from 22% to 33.14% and recognition accuracy from 18.13% to 39.44%. These results over multiple interactions are visualized in Figure S8(b, c).

Our results suggest that a pure computing approach, reminiscent of active learning (1), is not sufficient when deploying machine learning systems to people. In Figure S8(a), we qualitatively examine the the questions generated for the same image as the two agents train. When using only the knowledge reward, we find that the agent generates long, difficult-to-answer questions that are difficult for the recognition model. For example, "What is the thing the person is holding in their left hand?" require multiple reasoning steps: locating the person, finding their left hand, locating the object being held and then classifying the object. Consistent with prior work, we hypothesize that people on social media also prefer answering shorter questions, resulting in a drop in response rate (120).

13. Was the interaction representation required?

Language interactions impose a combinatorial search space for any conversational agent to navigate. In our experiments, we use recent advances in machine learning to learn an interaction representation (29, 31), identifying a lower-dimensional space where useful questions are likely to lie. We then use this interaction representation as a surrogate action space for the socially situated agent. We compare this approach against a more straight forward baseline that uses reinforcement learning to generate questions one token at a time (25, 40). This comparison studies whether using a lower dimensional surrogate space is necessary for learning from language interactions with people on social media.

Experimental setup. We compare two versions of our agent. One that uses the interaction representation space and a baseline version that uses the entire vocabulary space as its action space and generates a sequence of tokens that we stitch together to form a question. Both versions use both the interaction and the knowledge rewards. We launch both versions on social media during the same 8 month deployment. Each agent's rewards and interaction policy are updated after every

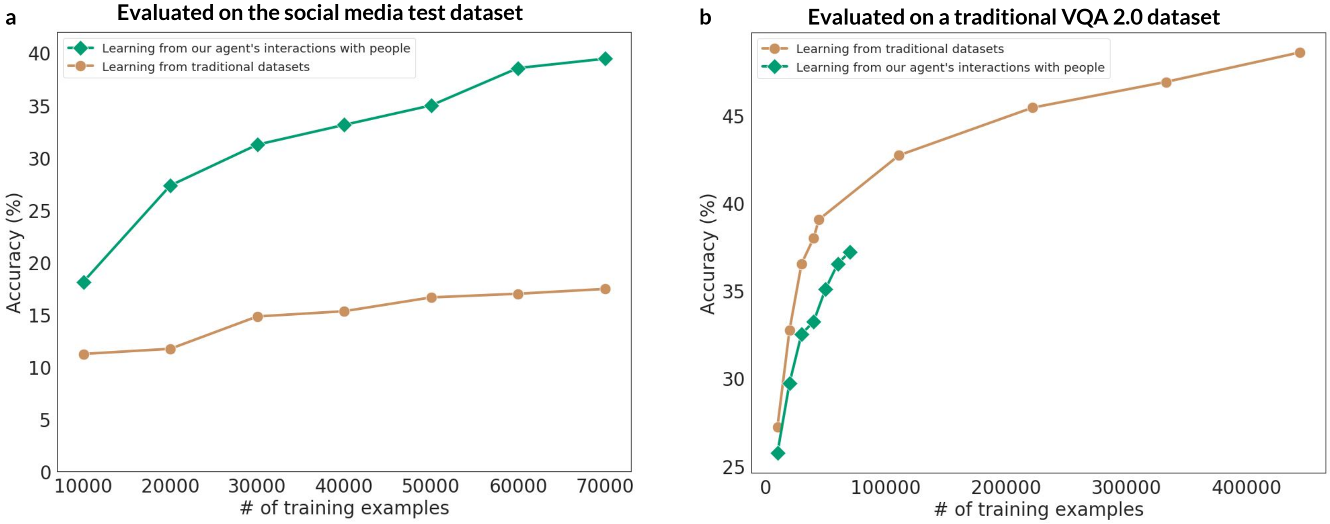

Fig. S7. We train two recognition models: one with training examples collected by our agent from interactions with people on social media, and another with training data from the large-scale traditional VQA 2.0 dataset. **(a)** We report the results of these two models when evaluated on the test dataset collected from social media. This evaluation demonstrates that our agent is capable of learning visual concepts that lead to higher accuracy on real world images people upload online. **(b)** We also report the results of these two models when evaluated on the standard VQA 2.0 benchmark dataset. This evaluation shows that even though there is a distribution shift, our model continues to improve as it continues to interact with more people

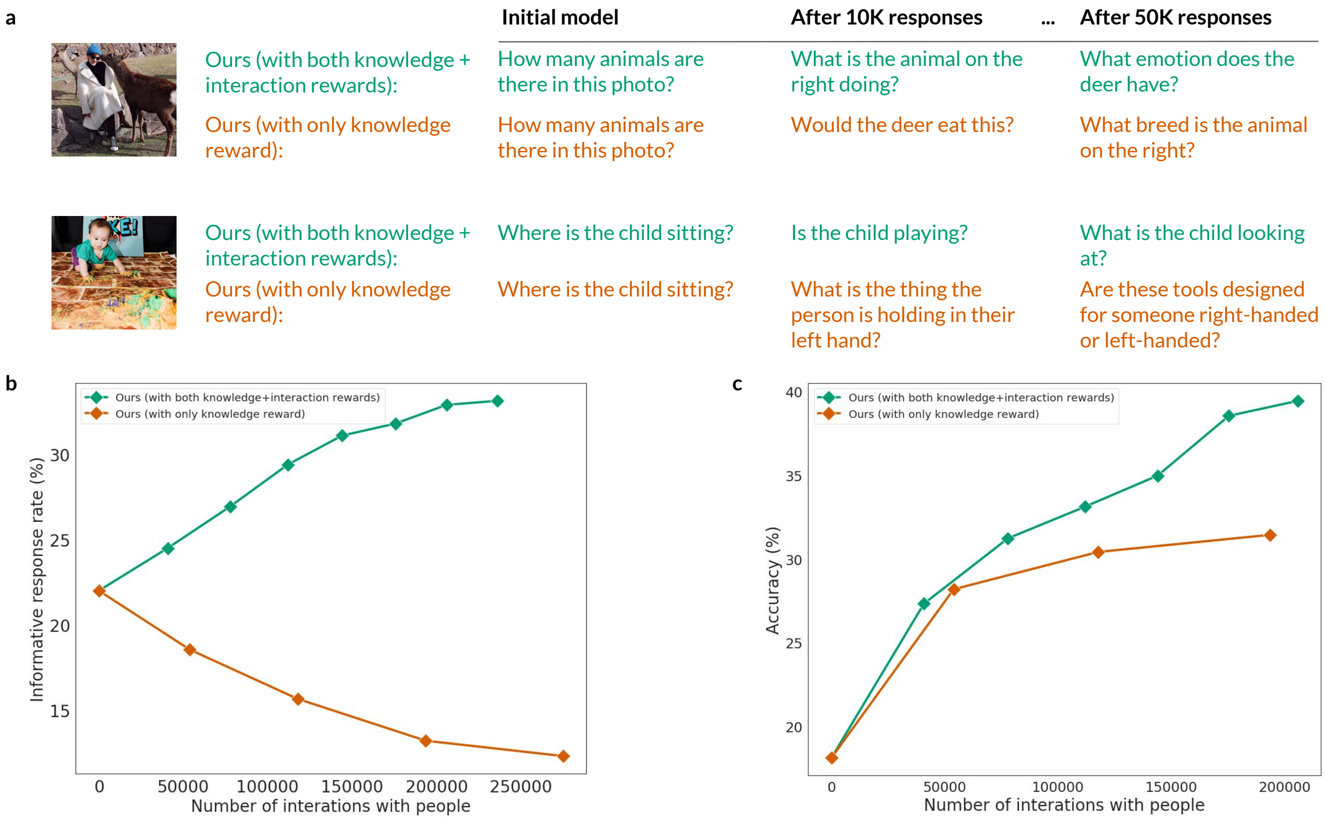

Fig. S8. We perform an ablation experiment where we compare our agent, which utilizes both the interaction and the knowledge reward with a variant — an agent that only uses the knowledge reward. This experiment demonstrates that a pure active learning objective is not sufficient when deploying machine learning systems that interact with people. **(a)** We show examples of questions generated by our agent versus ones generated by the variant . The variant generates longer and harder questions that require multiple reasoning steps. In the second image example, "What is the thing the person is holding in their left hand?" requires locating the person, their left hand, the relationship holding, and identifying an object. Meanwhile, "Are these tools designed for someone left-handed or right-handed?" requires knowing commonsense knowledge about what makes a tool left- or right-handed. Machine learning models today have difficulty answering such questions. Our experiment also indicates that people are less interested in answering long questions that require multiple reasoning steps. **(b)** Both agents are deployed on social media and interact with over $200,000$ each. We observe that the variant's response rate drops from 22% to 12.3%. When using both rewards, response rate increases from 22% to 33%. **(c)** We observe that using both rewards leads to faster improvements in recognition accuracy. The variant improves at a slower rate as fewer people responds to it.

10,000 interactions. To further strengthen the baseline, we add an extra language modeling reward (137), which incentivizes the generation of grammatically coherent questions, like the ones in existing datasets.

Metrics. We measure progress towards the two goals using the same metrics as the rest of the paper: (1) informative response rate measures the percentage of interactions that are relevant to the image, answerable and receive an answer, and (2) accuracy measures the percentage of questions that the recognition model answers correctly in our social media test dataset.

Results. Unfortunately, small updates to the policy's weights modify the baseline agent's behavior to generate meaningless sequences of tokens, causing responses rates to decrease, producing a vicious cycle where the agent cannot identify the small subset of actions that increase its reward. Its informative response rate quickly degenerates from an initial 22% to 6%, at which point, we decommissioned its deployment. In comparison, the agent that uses the interaction representation increases its informative response rate to 33% across interactions with 236,000 people (Figure S9(b)).

The baseline did not improve its ability to recognize new visual concepts; in fact, it only received 20,000 responses, most of them at the beginning of the deployment. In comparison, the agent with the interaction representation received 70,000 responses and improved accuracy on the test set from 18.13% to 39.44% (Figure S9(c)).

Discussion. Our results suggest that a pure language based reinforcement learning approach is difficult to tune, especially when the underlying reward function is consistently changing in reaction to improvements of the underlying vision model. The baseline approach quickly begins producing meaningless behavior, a result also identified by others (138). While we constrain the action space by learning a representation from questions in existing datasets, such a dataset of interactions might not be present for other socially situated instantiations in other domains. The challenges associated with developing compact, holistic representations is still an ongoing research topic (139) and should also be explored in the context of socially situated learning.

14. Would people respond differently to requests from another person instead of an AI agent?

In this section, we compare people's responses to our agent when it self-identifies as an automated agent versus when it doesn't. Before posting any question, our agent self-identifies itself with the following description: "We are a computer science research project." Our decision to self-identify is aimed to promote ethical transparency (see Section 15). However, the words or metaphors we use to self-identify can influence people's expectations of our agent and therefore encourage either pro- or anti-social behavior (140). Recent work demonstrated that the words or metaphors projecting high or low competence and warmth have a causal impact on end-user evaluations and behaviors (140). To minimize the effect of such metaphors, our agent's description was designed to minimize any interpretations of competence and warmth. For instance, we avoided descriptions that projected high competence (e.g. "We are an AI research project"), or high warmth (e.g. "We are a Computer Science project trying to learn about the world"). While a finer grained study of the impact of different descriptions is out of scope for our study, we do evaluate whether the presence of a description itself impacts the interactions.

Experimental setup. We study the effects of self-identifying by comparing against a non-disclosing condition, in which our agent does not self-identify; i.e. our agent only posts the question and does not post the self-identifying description. However, to promote transparency, people are sent a private message summarizing the purpose of the study 48 hours after the interaction, regardless of whether they respond. People who respond to this condition do not know that they were interacting with an automated agent until after they receive the private message.

Our agent interacted with a total of 3000 people without self-identifying. These 3000 interactions were uniformly spread out over the first $30K$ responses that the agent received. So, $\mathcal{V}$ interacted with 1000 people without self-identifying. The next 1000 interactions occurred using $\mathcal{V}$ and the last 1000 interactions were initiated by $\mathcal{V}$. By testing across multiple training updates, we are able to compare the effects of self-disclosing as the agent becomes more socially capable.

Results. We found no difference in people's responses between the two conditions. Both self-disclosing and not disclosing conditions received similar informative response rates across all three training steps. A Welsh's t-test concluded no statistical significance on informative response rate, regardless of whether our agent self-disclosed, across all three training steps: ($t(46452) = -0.36, p < 0.71$), ($t(41814) = -0.63, p < 0.52$), and ($t(38103) = -0.61, p < 0.54$).

Discussion. These results suggest that people interact with our agent, not because it is an automated AI agent, but because they are wiling to respond even if another person were to post those particular questions. Future work should study how the descriptors attached to agents not only changes human expectations but whether it alters the types of responses and learning dynamics of socially situated learning.

15. Ethical considerations

As machines are deployed and learn to socially interact with people in existing social environments, it is important to reflect on the potential negative implications of computing research, and how they can be addressed. There are numerous sources of risk associated with experiments that involve human-AI interaction. Such risks can be exemplified when alloyed with biases inherited from pretrained machine learning models and datasets.(141) In this section, we lay out some of the risks to the population of people our experiments involved, the principles we followed, and how we mitigated the risks. We also discuss potential implications of socially situated AI research to the broader society at large.

Which social media posts should agents interact with? Our agent interacted with a sizable population of people on a social network throughout its deployment. To avoid interacting with private content, we made certain that all our interactions were with publicly accessible posts; we only interacted with content that could be viewed with a publicly accessible URL. Also, we only interacted with public posts that contained one of the common hashtags that users of the platform apply to improve

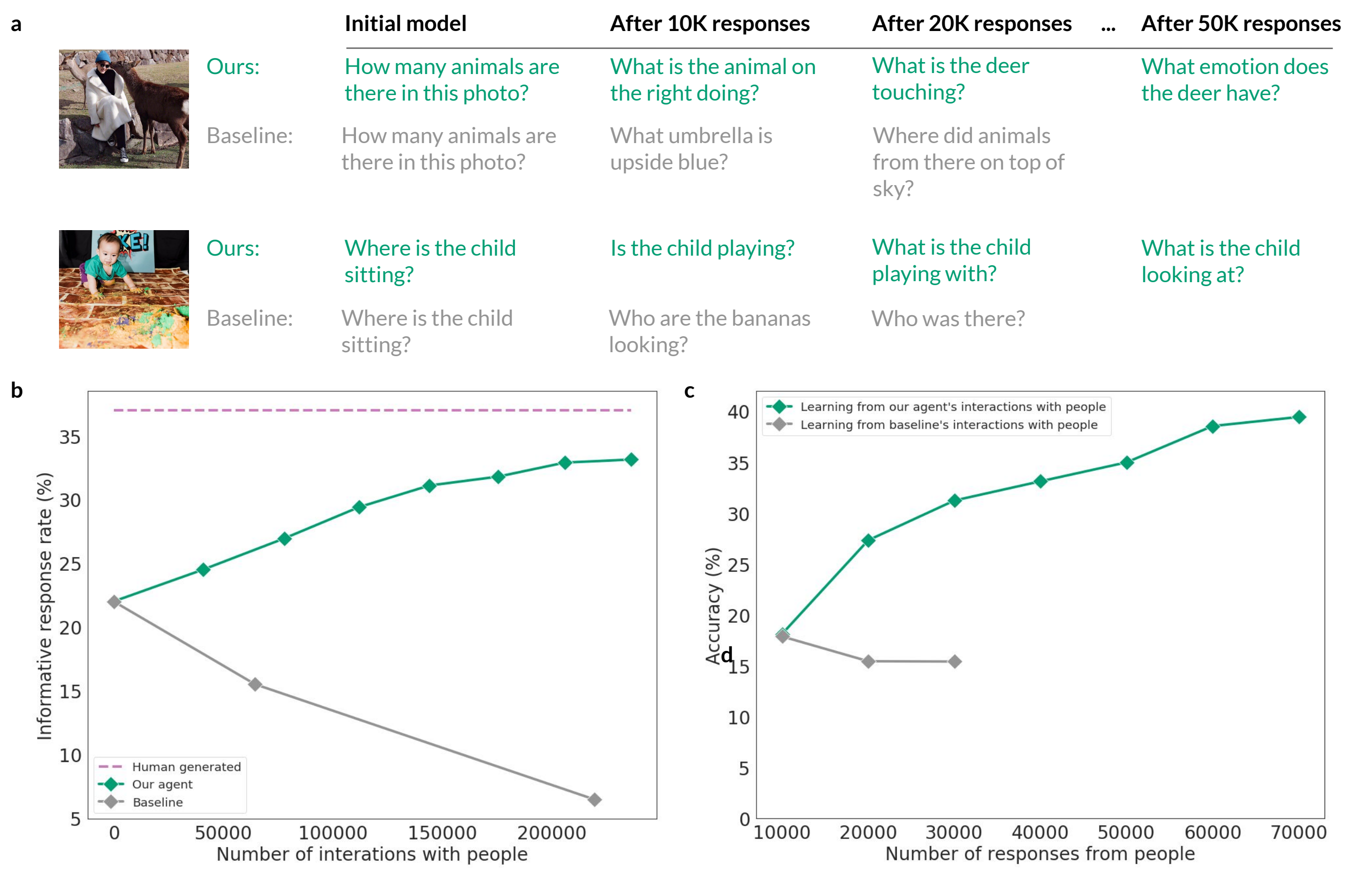

Fig. S9. We show changes in behavior, informative response rate, and recognition accuracy as agents interact with people and gather new visual knowledge. **(a)** Top row: the agent using the interaction representation as an action space learns that people don't like answering counting questions and moves on to asking about the "animal" and later refers to the animal as a "deer". Bottom row: this agent first learns whether a child is playing and later starts to ask about what the child is interacting with. In comparison, the baseline approach quickly begins generating incoherent questions. **(b)** We plot informative response rate versus the number of interactions initiated. While the agent learns to increase its response rate by 50%, the baseline method degenerates by 72%. In comparison, questions written by paid workers achieve a 37% response rate. **(c)** We visualize visual accuracy versus the number of responses from people. Our agents learns recognize new concepts while the baseline is unable to improve.

discoverability, clickthrough rate, and engagement. We advocate for future interactions with online media to follow similar guidelines: only interact with public content that people indicate as accepting of engagement.

How should AI agents self-identify? We ensured that our agent introduced itself before asking a question by stating that "We are a computer science research project." This message was also repeated on our agent's profile biography, ensuring that people who encounter our profile were always aware of the purpose of the account and its interactions. To promote transparency, we sent people a private message summarizing the purpose of the study after every interaction, whether people responded or not (142). We also provided them with our email address, should they have any concerns or want to remove their data from our experiments (143). Finally, we also hired paid crowd workers to moderate the questions we wanted to post by verifying that our questions could not be construed as rude or malicious. We believe that any AI agents, whether in research or products, should self-identify before starting any human interaction. In cases where the self-identification interferes with the research study, we advocate studies to moderate the interaction, self-identify after the interaction, and provide people with the option of removing their data from the interaction. Furthermore, because of the potential for harm, we advocate the use of workers or the researchers themselves to vet content if feasible before it is posted during a research deployment.

What are the risks for paid annotators? Paid workers from Amazon Mechanical Turk were critical for our experiments. They annotated social media images to create a test set for evaluation, annotated images to help filter out memes and cartoons, verified that questions we posted would not be interpreted as rude. Recent work has highlighted negative side effects caused by asking workers to annotate or moderate large volumes of data or annotating potentially abusive or harmful content (144). We mitigate these problems by paying our workers a fair wage of $12-$15 an hour, which is higher than the current average minimum wage of $7.25 in the United States of America (145). Additionally, we limited the number of interactions we initiate, and thus, the number of interaction moderation tasks to 3000 per day. Finally, since we source our images from a social media platform that polices its content, the images were less likely to contain abusive or harmful content.

What are the risks associated with diverting people's attention? Another risk worth considering is the ethical calculus behind diverting the attention hundreds of thousands of people to answer our questions. Throughout our deployments, we monitored how people reacted to our agent to justify its deployment. Aside from answering our questions, many responses contained gratitude or positive reactions (e.g. ":D" or "thank you for asking") and positive valence emojis. In contrast, when we deployed existing reinforcement learning algorithms as our baseline, we noticed a decrease in response rate. We stopped the baseline model once response rate dropped from 22% to 6%. Future work developing human interaction should similarly measure whether how people are reacting, or if they are reacting voluntarily at all.

What are the biases inherited from past datasets? Our interaction representation was trained using readily available data from previously collected Computer Vision datasets (27, 28). The questions contained in these datasets might be interpreted as inappropriate when posted to social media. For example, some questions ask about people's emotional states (e.g. "is the woman happy?"), age (e.g. "how old is the boy?"), and assume occupation (e.g."what is the construction worker holding?"). We removed any question that could be interpreted as malicious or subjective by manually inspecting every question in the dataset used to initialize our agent's question generation components. Also, there are explicit assumptions of people's genders and sexuality throughout the dataset. To avoid discrimination with regards to gender, occupation, and age (141), we replaced all explicit mentions of words such as "woman", "boy", "construction worker", etc. with "person". Even with our changes, there is still an inherit image content bias inherited from these datasets, which sourced its images using a set of 91 object categories (146).

What are the biases promoted by our data collection process? Our deployment is meant to demonstrate the possibility of social situated AI agents that can learn from interactions with people. Our instantiation of this possibility, through a social media deployment, is accompanied by a host of potential biases. Social media users represent a specific portion of society and these platforms promote the curation of concepts that are visually appealing but unrepresentative of concepts that people encounter in the real world (147). We induce further biases by polling images that contain a fixed number of hashtags, and utilize a filtering model to remove unwanted cartoons or memes. We also do not have any demographic information about the social media users we interact with and could be specifically receiving responses from a particularly active subgroup on social media. Our agent may have learned culture specific topics to ask questions about. There are numerous studies that have identified the negative effects of deploying AI systems that interact with demographics that were absent in their training (141, 148). Multi-year longitudinal studies need to evaluate how agent's emergent behaviors might learn to favor interactions deemed acceptable by the majority, at the cost of marginalizing others.

What are the privacy risks with collecting data through AI social interactions? Users are increasingly sharing their images on various social networks, including Facebook, Instagram, TikTok, and Flickr. Social networks can be one of the riskiest personal information leakage sources (149). Shared images can reveal sensitive information about people, their geolocations, interactivity and relationships, as well as their check-ins, activities, and food preferences (150–153). Companies can gather this information to train models that can infer user preference and send targeted ads (154, 155). Such invasive methods passively gather information: they extract private information from data that people have already posted online. But with a framework for socially situated AI, newer methods can be developed to actively elicit responses to reveal information that is unavailable online. As such, there is a need to develop new automated privacy management mechanisms to support users in protecting their privacy.

In order to protect users against existing data privacy leaks, a handful of privacy management systems have already been proposed: iPrivacy (156) notifies users if the image they want to post contains objects that might reveal private information. Visual Privacy Advisor (157) classifies personal information in images into 68 attributes, which users can customize as alerts,

which inform them if their post contains one of those attributes. Warned by such alerts, users can decide not to post their image; alternatively, they can invoke automatic redaction methods to transform (158) or obfuscate private information (159, 160).

When eliciting information through social interactions, we advocate that future interactive AI systems inform users of each data point that would be incorporated into a training dataset. Users should be provided with contact information or a procedure that allows them to withdraw their data from such a curation. In our deployment, we informed users that their answers would be utilized to improve our system's visual recognition ability and provided them with contact information to remove their responses.

How to release/license datasets collected using social interactions? Aside from informing users that their data would be added to a training dataset, privacy preservation processing is also required when the data custodians publicly release the data. Database release is critical to promote reproducibility in machine learning research. However, releasing a dataset collected through social interactions could exacerbate privacy-threatening annotations. A future research direction should explore methods to generate synthetic data that retains the statistical properties of the real data while reducing the risk of information disclosure (161). The recent success of using synthetic training data produced from a pre-trained generative model demonstrates the promise of replacing real datasets with synthetic ones created using generative models (162). This result suggests the possibility of training a private generative model on an unreleased socially collected dataset and performing experiments with the synthetic data produced by this said model. Experimental results, along with the synthetic dataset could be released to facilitate reproducibility while minimizing privacy leaks. We plan to explore such methods in order to safely release our current and future socially acquired datasets.

What are the risks associated with malicious usage of our technology? Learning people's interaction preferences could be used as a manipulation strategy to induce behavioral changes (18). We are, and should be, concerned, about malicious actors teaching such a agent how to manipulate people online, for example inducing emotional or affective shifts (163) or inducing anti-social behavior (164). A goal of modeling human interest can also itself be problematic: many social media platforms are transitioning from a focus on short term engagement metrics to longer-term ones, as they believe such metrics will lead to longer term community and platform health. We designed our agent toward creating enjoyable interactions online—largely identifying topics of interest to ask questions about, rather than manipulative forms of asking—and we believe that it is important that future work also be transparent about what goals it is optimizing and what rewards it responds to.

What are the risks associated with a society where interpersonal communication is augmented with AI? With simple functions like email autocomplete to complex applications like Google Duplex, we are entering an era of AI-mediated communication where interpersonal communication is augmented and even generated by AI. Our work situates agents in a social environment, i.e. social network, which is predominantly occupied by people. Recent studies indicate that when people interact with a mixed set of AI- and human-written profiles, they mistrusted those whose profiles were labeled as or suspected to be written by AI (165). To improve trust in a social-technical system and promote pro-social behavior, policies should be enacted to enforce transparent self-identification of AI-agumentations and generation, creating a clear distinction when interacting with people versus machines.

What are the risks associated with job displacement? AI systems displacing jobs is becoming increasingly a matter of concern as recent Machine Learning breakthroughs have attracted public attention (166, 167). This attention has also catalyzed the rise of conversational bots over the last few years (168). Our work, which promotes learning from human interaction, can be used to automate tasks, such as customer service, which have traditionally been regarded as hard to automate (143, 169–171).

16. Limitations and further work

These results suggest the potential for developing AI agents that can learn from social interactions with people while simultaneously modeling and supporting human interests. Our deployment is meant to serve as a prototype; as such, there exists numerous avenues for improvement. We lay out some critical limitations and opportunities for future work in this section.

First, if our agent posts questions irrelevant to the image, people often answer an interpretation of the originally incorrect question. For example, we might ask, "What is the cat doing?", to an image without a cat. Instead of ignoring the question, people reply by correcting the animal in the photo and then answer the question: "You mean the goat? It is trying to climb a tree." Past literature explains that people prefer to suggest alternative framings and even modify the information requested to fit the context (172). We currently classify these responses as confused or irrelevant to the image; this is far too simple a model. Instead, accounting for such responses could not only provide additional signal about what topics people naturally gravitate towards, it would also help re-train the question generation model to produce more relevant questions.

Second, people are more likely to respond when the reason behind asking a question is explained (18, 173). Providing a transparent (and honest) reason, along with the question can establish a social cadence with the person and lead to more natural interactions (174, 175). Our interactions are currently limited to one question and answer, so future work should explore the impact of longer dialogues sequences (176). Related, it is important to consider how agents can provide direct value to the people they interact with, establishing a mutually beneficial relationship.

Third, Computer Vision has found it expensive to build fine-grained datasets where annotators with domain expertise need to be hired, and paid annotators often do not offer the expertise (177). Learning from interactions with people might mitigate this problem by modeling user interests and seeking to engage users with respect to their unique skills (128). For example, someone uploading pictures of a flower is likely to have seen that flower in the real world and so, more likely to know the names of the flora.

Fourth, any social media platform will bias people to post pictures of a specific aesthetic appeal.* As such, future work should also carefully consider the biases associated with specific media sources. We discuss these biases in Section 15.

To reduce the combinatorially vast search space, we freeze the decoder's parameters once it is initialized from existing datasets. Interactions with people are only used to re-trains the agent's policy to choose better latent categories within the interaction representation. While this decision allows us to learn from sparse interactions and avoid searching over large action spaces, it comes at a cost: because the decoder weights are pretrained using readily available data, it never learns to generate questions on new topics. Future work should explore mechanisms to navigate the combinatorial space without requiring the agent to limit the scope of its interactions. This is particularly vital as agents learn new concepts; learning to ask followup questions is beyond the scope of our paper but should be explored in future work; followup questions will need to reference the new concepts elicited from past dialogue turns.

While we deploy our agent on one social media platform, we also ran experiments on numerous other platforms. We chose our social media platform for its centralized focus on visual content. We found very rare < 10 total instances of users replying with negative or sarcastic comments. On some other platforms, however, we found the opposite to be true — 90% of responses were sarcastic, confirming the findings of a previously released conversational agent called Tay (178, 179) Unlike Tay, however, users do not initiate interactions; our agent chooses interesting images and questions and actively reaches out to users, reducing the likelihood of a planned attacked. However, it seems clear that future work should incorporate user trust when interfacing with people.

Advances in dialogue generation have been attempting to curate natural language generation to be more "meaningful" or "natural" for human users(32, 180, 181). In contrast, our questions are neither designed to be meaningful, nor human-like. They are task-driven interactions that attempt to elicit useful information. As long as people respond to them, our evaluation metrics demonstrate the utility of those interactions. That being said, long-term interactions with people might need to evaluate how human-like an interaction is. Future work should expand upon our limited definition of what constitutes a social interaction to incorporate measures such as fluency, inquisitiveness, interestingness, and humanness (181).

Our deployment explores the utility of social situated learning through the context of visual question answering (28). As already mentioned, natural language question answering generalizes many computer vision tasks: object detection (e.g., "What is in the image?"), fine-grained recognition (e.g. "What kind of flowers are in the vase?"), attribute classification (e.g., "What material is the table made of?"), knowledge base reasoning (e.g., "Is this a food vegetarian?"), and commonsense reasoning (e.g., "Was this taken in the winter?"). However, the results from our deployment are insufficient to suggest that similar techniques would transfer to non-computer vision tasks or to interactions beyond a single turn of question answering. For instance, robotic tasks might require interacting with people through motion; healthcare applications might require generating novel questions when encountering with rare medical conditions; next-generation multimedia and creativity tools could require simultaneous multi-modal interactions. Even though our socially situated AI framework generalizes to all these applications, our experiments explore only one specific computer vision instantiation. Future work could utilize the framework to contextualize new domains where socially situated learning could be beneficial and identify the domain-specific technical challenges in each domain.

Recently, transfer learning has become the norm (182). The choice of which pre-training method and dataset achieves the most general features is still undecided. As such, our experiments pre-trained only using the standard ImageNet dataset (19). We did not pre-train the computer vision model on visual question answering datasets like VQA 2.0 (28) or Visual Genome (27). The purpose of our experiments was to showcase that models *could* learn from their interactions with people and not necessarily benchmark how much improvement is possible when starting with a pre-trained model. Future work should establish standardized benchmarks, pre-training data, and test sets for open-ended visual question answering and for socially situated learning more broadly.

One of the largest limitations of our work—and for all machine learning solutions that interface with humans—is the subjective inconsistency of human behavior. Progress in machine learning relies on consistent evaluation metrics and training procedures. Introducing humans into the evaluation pipeline has only seen success in particular scenarios where psychophysics-inspired experimental design made it possible to extract consistent human behavior (183). To evaluate our deployment, we measured how often people provided our agent with new information. We attempted to remove biases in our evaluation by evaluating our agent slowly over multiple months. However, our reported values might not be consistent when restricted to specific demographics of users, especially to those people who do not engage in conversations with others on social networks. An ideal socially situated evaluation scheme should take a more holistic approach, measuring how agents interact with various demographics. Our future work plan is to develop benchmarks with consistent evaluation protocols, even if those protocols involve human interactions during training or testing.

References

1. B Settles, Active learning literature survey, (University of Wisconsin-Madison Department of Computer Sciences), Technical report (2009).
2. A Siddhant, ZC Lipton, Deep bayesian active learning for natural language processing: Results of a large-scale empirical study in *Empirical Methods in Natural Language Processing (EMNLP)*. (2018).
3. D Lowell, ZC Lipton, BC Wallace, Practical obstacles to deploying active learning in *Empirical Methods in Natural Language Processing (EMNLP)*. (2019).

* See Emma Sheffer's curation of Instagram posts by semantic and aesthetic similarity @insta_repeat

4. Y Deng, K Chen, Y Shen, H Jin, Adversarial active learning for sequences labeling and generation in *International Joint Conference on Artificial Intelligence (IJCAI).* pp. 4012–4018 (2018).
5. Y Gal, R Islam, Z Ghahramani, Deep bayesian active learning with image data in *International Conference on Machine Learning (ICML).* (2017).
6. Y Shen, H Yun, ZC Lipton, Y Kronrod, A Anandkumar, Deep active learning for named entity recognition in *Proceedings of the Second Workshop on Representation Learning for NLP (Repl4NLP).* (2017).
7. I Misra, et al., Learning by asking questions in *2018 IEEE/CVF Conference on Computer Vision and Pattern Recognition.* (IEEE), pp. 11–20 (2018).
8. Y Yang, M Loog, Active learning using uncertainty information in *2016 23rd International Conference on Pattern Recognition (ICPR).* (IEEE), pp. 2646–2651 (2016).
9. A Kapoor, K Grauman, R Urtasun, T Darrell, Active learning with gaussian processes for object categorization in *2007 IEEE 11th International Conference on Computer Vision.* (IEEE), pp. 1–8 (2007).
10. A Freytag, E Rodner, J Denzler, Selecting influential examples: Active learning with expected model output changes in *European Conference on Computer Vision.* (Springer), pp. 562–577 (2014).
11. Y Abramson, Y Freund, Active learning for visual object recognition, (University of California, San Diego), Technical report (2004).
12. B Collins, J Deng, K Li, L Fei-Fei, Towards scalable dataset construction: An active learning approach in *European Conference on Computer Vision (ECCV).* pp. 86–98 (2008).
13. AJ Joshi, F Porikli, N Papanikolopoulos, Multi-class active learning for image classification in *2009 IEEE Conference on Computer Vision and Pattern Recognition.* (IEEE), pp. 2372–2379 (2009).
14. Y Gal, Z Ghahramani, Dropout as a Bayesian approximation: Representing model uncertainty in deep learning in *International Conference on Machine Learning (ICML).* (2016).
15. A Kendall, Y Gal, What uncertainties do we need in Bayesian deep learning for computer vision? in *Advances in Neural Information Processing Systems (NeurIPS).* pp. 5574–5584 (2017).
16. N Houlsby, F Huszár, Z Ghahramani, M Lengyel, Bayesian active learning for classification and preference learning. *ArXiv* **abs/1112.5745** (2011).
17. O Sener, S Savarese, Active learning for convolutional neural networks: A core-set approach in *International Conference on Learning Representations (ICLR).* (2018).
18. RB Cialdini, RB Cialdini, *Influence: The psychology of persuasion.* (Collins New York), (2007).
19. J Deng, et al., Imagenet: A large-scale hierarchical image database in *IEEE Conference on Computer Vision and Pattern Recognition.* (IEEE), pp. 248–255 (2009).
20. K Healy, A Schussman, The ecology of open-source software development, (Technical report, University of Arizona, USA), Technical report (2003).
21. BM Hill, Almost wikipedia: Eight early encyclopedia projects and the mechanisms of collective action, Technical report (2013).
22. J Reich, R Murnane, J Willett, The state of wiki usage in us k–12 schools: Leveraging web 2.0 data warehouses to assess quality and equity in online learning environments. *Educ. Res.* **41**, 7–15 (2012).
23. Z Yang, X He, J Gao, L Deng, A Smola, Stacked attention networks for image question answering in *Proceedings of the IEEE conference on computer vision and pattern recognition.* pp. 21–29 (2016).
24. J Pennington, R Socher, C Manning, Glove: Global vectors for word representation in *Proceedings of the 2014 conference on empirical methods in natural language processing (EMNLP).* pp. 1532–1543 (2014).
25. J Li, et al., Deep reinforcement learning for dialogue generation in *Proceedings of the 2016 conference on empirical methods in natural language processing (EMNLP).* (2016).
26. T Zhao, K Xie, M Eskenazi, Rethinking action spaces for reinforcement learning in end-to-end dialog agents with latent variable models (2019).
27. R Krishna, et al., Visual genome: Connecting language and vision using crowdsourced dense image annotations. *Int. J. Comput. Vis.* **123**, 32–73 (2017).
28. S Antol, et al., Vqa: Visual question answering in *Proceedings of the IEEE International Conference on Computer Vision.* pp. 2425–2433 (2015).
29. R Krishna, M Bernstein, L Fei-Fei, Information maximizing visual question generation in *IEEE Conference on Computer Vision and Pattern Recognition.* (2019).
30. SR Bowman, et al., Generating sentences from a continuous space in *Proceedings of The 20th SIGNLL Conference on Computational Natural Language Learning.* (Association for Computational Linguistics, Berlin, Germany), pp. 10–21 (2016).
31. A van den Oord, O Vinyals, K Kavukcuoglu, Neural discrete representation learning in *NIPS.* (2017).
32. I Serban, A Sordoni, Y Bengio, A Courville, J Pineau, Building end-to-end dialogue systems using generative hierarchical neural network models in *Proceedings of the AAAI Conference on Artificial Intelligence.* Vol. 30, (2016).
33. T Zhao, M Eskenazi, Zero-shot dialog generation with cross-domain latent actions in *Proceedings of the 19th Annual SIGdial Meeting on Discourse and Dialogue.* (Association for Computational Linguistics, Melbourne, Australia), pp. 1–10 (2018).
34. E Jang, S Gu, B Poole, Categorical reparameterization with gumbel-softmax (2016).

35. S Hochreiter, J Schmidhuber, Long short-term memory. *Neural computation* **9**, 1735–1780 (1997).
36. U Jain, Z Zhang, AG Schwing, Creativity: Generating diverse questions using variational autoencoders. in *Proceedings of the IEEE Conference on Computer Vision and Pattern Recognition.* pp. 5415–5424 (2017).
37. MT Luong, H Pham, CD Manning, Effective approaches to attention-based neural machine translation (2015).
38. A Vaswani, et al., Attention is all you need in *Advances in neural information processing systems.* pp. 5998–6008 (2017).
39. J Devlin, MW Chang, K Lee, K Toutanova, Bert: Pre-training of deep bidirectional transformers for language understanding (2018).
40. J Schulman, F Wolski, P Dhariwal, A Radford, O Klimov, Proximal policy optimization algorithms (2017).
41. M Hausknecht, P Stone, Deep recurrent q-learning for partially observable mdps in *AAAI Fall Symposium on Sequential Decision Making for Intelligent Agents.* (2015).
42. V Mnih, et al., Human-level control through deep reinforcement learning. *nature* **518**, 529–533 (2015).
43. ME Taylor, A Borealis, Improving reinforcement learning with human input in *IJCAI.* pp. 5724–5728 (2018).
44. AL Thomaz, C Breazeal, Teachable robots: Understanding human teaching behavior to build more effective robot learners. *Artif. Intell.* **172**, 716–737 (2008).
45. WB Knox, P Stone, Learning non-myopically from human-generated reward in *Proceedings of the 2013 international conference on Intelligent user interfaces.* (ACM), pp. 191–202 (2013).
46. R Loftin, et al., Learning behaviors via human-delivered discrete feedback: modeling implicit feedback strategies to speed up learning. *Auton. agents multi-agent systems* **30**, 30–59 (2016).
47. AL Thomaz, C Breazeal, , et al., Reinforcement learning with human teachers: Evidence of feedback and guidance with implications for learning performance in *Aaai.* (Boston, MA), Vol. 6, pp. 1000–1005 (2006).
48. Z Wang, ME Taylor, Improving reinforcement learning with confidence-based demonstrations. in *IJCAI.* pp. 3027–3033 (2017).
49. B Peng, et al., A need for speed: Adapting agent action speed to improve task learning from non-expert humans in *Proceedings of the 2016 International Conference on Autonomous Agents & Multiagent Systems.* (International Foundation for Autonomous Agents and Multiagent Systems), pp. 957–965 (2016).
50. CL Isbell, et al., Cobot in lambdamoo: An adaptive social statistics agent. *Auton. Agents Multi-Agent Syst.* **13**, 327–354 (2006).
51. C Isbell, CR Shelton, M Kearns, S Singh, P Stone, A social reinforcement learning agent in *Proceedings of the fifth international conference on Autonomous agents.* (ACM), pp. 377–384 (2001).
52. WB Knox, P Stone, Interactively shaping agents via human reinforcement: The tamer framework in *Proceedings of the fifth international conference on Knowledge capture.* (ACM), pp. 9–16 (2009).
53. IV Serban, et al., A hierarchical latent variable encoder-decoder model for generating dialogues in *Proceedings of the Thirty-First AAAI Conference on Artificial Intelligence.* pp. 3295–3301 (2017).
54. X Du, J Shao, C Cardie, Learning to ask: Neural question generation for reading comprehension in *Proceedings of 55th annual meeting of the association for computational linguistics.* (2017).
55. J Yang, J Lu, S Lee, D Batra, D Parikh, Visual curiosity: Learning to ask questions to learn visual recognition in *2nd Conference on Robot Learning.* (2018).
56. AK Vijayakumar, et al., Diverse beam search: Decoding diverse solutions from neural sequence models in *Thirty-Second AAAI Conference on Artificial Intelligence.* (2018).
57. N Mostafazadeh, et al., Generating natural questions about an image in *Proceedings of the 54th Annual Meeting of the Association for Computational Linguistics.* (2016).
58. M Ren, R Kiros, R Zemel, Exploring models and data for image question answering in *Advances in neural information processing systems.* pp. 2953–2961 (2015).
59. S Vijayanarasimhan, K Grauman, Large-scale live active learning: Training object detectors with crawled data and crowds. *Int. J. Comput. Vis.* **108**, 97–114 (2014).
60. GJ Qi, XS Hua, Y Rui, J Tang, HJ Zhang, Two-dimensional active learning for image classification in *2008 IEEE Conference on Computer Vision and Pattern Recognition.* (IEEE), pp. 1–8 (2008).
61. M Cakmak, C Chao, AL Thomaz, Designing interactions for robot active learners. *IEEE Transactions on Auton. Mental Dev.* **2**, 108–118 (2010).
62. M Cakmak, AL Thomaz, Optimality of human teachers for robot learners in *2010 IEEE 9th International Conference on Development and Learning.* (IEEE), pp. 64–69 (2010).
63. A Guillory, JA Bilmes, Simultaneous learning and covering with adversarial noise. in *ICML.* Vol. 11, pp. 369–376 (2011).
64. S Thrun, Is learning the n-th thing any easier than learning the first? in *Advances in neural information processing systems.* pp. 640–646 (1996).
65. J Kirkpatrick, et al., Overcoming catastrophic forgetting in neural networks. *Proc. national academy sciences* **114**, 3521–3526 (2017).
66. P Ruvolo, E Eaton, Ella: An efficient lifelong learning algorithm in *International Conference on Machine Learning.* pp. 507–515 (2013).
67. Y Jia, JT Abbott, JL Austerweil, T Griffiths, T Darrell, Visual concept learning: Combining machine vision and bayesian generalization on concept hierarchies in *Advances in Neural Information Processing Systems.* pp. 1842–1850 (2013).
68. M Yatskar, V Ordonez, A Farhadi, Stating the obvious: Extracting visual common sense knowledge in *Proceedings of the*

2016 Conference of the North American Chapter of the Association for Computational Linguistics: Human Language Technologies. pp. 193–198 (2016).
69. SK Divvala, A Farhadi, C Guestrin, Learning everything about anything: Webly-supervised visual concept learning in *Proceedings of the IEEE Conference on Computer Vision and Pattern Recognition.* pp. 3270–3277 (2014).
70. A Carlson, et al., Toward an architecture for never-ending language learning. in *AAAI.* (Atlanta), Vol. 5, p. 3 (2010).
71. X Chen, A Shrivastava, A Gupta, Neil: Extracting visual knowledge from web data in *Computer Vision (ICCV), 2013 IEEE International Conference on.* (IEEE), pp. 1409–1416 (2013).
72. T Mitchell, et al., Never-ending learning. *Commun. ACM* **61**, 103–115 (2018).
73. N Chentanez, AG Barto, SP Singh, Intrinsically motivated reinforcement learning in *Advances in neural information processing systems.* pp. 1281–1288 (2005).
74. J Schmidhuber, Formal theory of creativity, fun, and intrinsic motivation (1990–2010). *IEEE Transactions on Auton. Mental Dev.* **2**, 230–247 (2010).
75. M Bellemare, et al., Unifying count-based exploration and intrinsic motivation in *Advances in neural information processing systems.* pp. 1471–1479 (2016).
76. J Fu, J Co-Reyes, S Levine, Ex2: Exploration with exemplar models for deep reinforcement learning in *Advances in neural information processing systems.* pp. 2577–2587 (2017).
77. J Gottlieb, PY Oudeyer, M Lopes, A Baranes, Information-seeking, curiosity, and attention: computational and neural mechanisms. *Trends cognitive sciences* **17**, 585–593 (2013).
78. R Houthooft, et al., Vime: Variational information maximizing exploration in *Advances in Neural Information Processing Systems.* pp. 1109–1117 (2016).
79. J Achiam, S Sastry, Surprise-based intrinsic motivation for deep reinforcement learning (2017).
80. D Pathak, P Agrawal, AA Efros, T Darrell, Curiosity-driven exploration by self-supervised prediction in *Proceedings of the IEEE Conference on Computer Vision and Pattern Recognition Workshops.* pp. 16–17 (2017).
81. BC Stadie, S Levine, P Abbeel, Incentivizing exploration in reinforcement learning with deep predictive models in *Neural Information Processing Systems, Deep RL Workshop.* (2015).
82. AH Qureshi, Y Nakamura, Y Yoshikawa, H Ishiguro, Intrinsically motivated reinforcement learning for human–robot interaction in the real-world. *Neural Networks* **107**, 23–33 (2018).
83. S Amershi, M Cakmak, WB Knox, T Kulesza, Power to the people: The role of humans in interactive machine learning. *AI Mag.* **35**, 105–120 (2014).
84. JA Fails, DR Olsen Jr, Interactive machine learning in *Proceedings of the 8th international conference on Intelligent user interfaces.* (ACM), pp. 39–45 (2003).
85. J Fogarty, D Tan, A Kapoor, S Winder, Cueflik: interactive concept learning in image search in *Proceedings of the sigchi conference on human factors in computing systems.* (ACM), pp. 29–38 (2008).
86. K Patel, J Fogarty, JA Landay, B Harrison, Investigating statistical machine learning as a tool for software development in *Proceedings of the SIGCHI Conference on Human Factors in Computing Systems.* (ACM), pp. 667–676 (2008).
87. K Patel, et al., Gestalt: integrated support for implementation and analysis in machine learning in *Proceedings of the 23nd annual ACM symposium on User interface software and technology.* (ACM), pp. 37–46 (2010).
88. JC Chang, S Amershi, E Kamar, Revolt: Collaborative crowdsourcing for labeling machine learning datasets in *Proceedings of the 2017 CHI Conference on Human Factors in Computing Systems.* (ACM), pp. 2334–2346 (2017).
89. S Amershi, J Fogarty, A Kapoor, D Tan, Examining multiple potential models in end-user interactive concept learning in *Proceedings of the SIGCHI Conference on Human Factors in Computing Systems.* (ACM), pp. 1357–1360 (2010).
90. X Yuan, et al., Machine comprehension by text-to-text neural question generation in *Proceedings of the 2nd Workshop on Representation Learning for NLP.* pp. 15–25 (2017).
91. R Fiebrink, PR Cook, D Trueman, Play-along mapping of musical controllers in *ICMC.* (Citeseer), (2009).
92. S Amershi, et al., Software engineering for machine learning: A case study in *2019 IEEE/ACM 41st International Conference on Software Engineering: Software Engineering in Practice (ICSE-SEIP).* (IEEE), pp. 291–300 (2019).
93. S Amershi, et al., Guidelines for human-ai interaction in *Proceedings of the 2019 CHI Conference on Human Factors in Computing Systems.* pp. 1–13 (2019).
94. T Winograd, What does it mean to understand language? *Cogn. science* **4**, 209–241 (1980).
95. THK Huang, JC Chang, JP Bigham, Evorus: A crowd-powered conversational assistant built to automate itself over time in *Proceedings of the 2018 CHI Conference on Human Factors in Computing Systems.* (ACM), p. 295 (2018).
96. WS Lasecki, et al., Chorus: a crowd-powered conversational assistant in *Proceedings of the 26th annual ACM symposium on User interface software and technology.* (ACM), pp. 151–162 (2013).
97. A Fourney, R Mann, M Terry, Query-feature graphs: bridging user vocabulary and system functionality in *Proceedings of the 24th annual ACM symposium on User interface software and technology.* (ACM), pp. 207–216 (2011).
98. K Vaccaro, S Shivakumar, Z Ding, K Karahalios, R Kumar, The elements of fashion style in *Proceedings of the 29th Annual Symposium on User Interface Software and Technology.* (ACM), pp. 777–785 (2016).
99. T Gao, M Dontcheva, E Adar, Z Liu, KG Karahalios, Datatone: Managing ambiguity in natural language interfaces for data visualization in *Proceedings of the 28th Annual ACM Symposium on User Interface Software & Technology.* (ACM), pp. 489–500 (2015).
100. SI Wang, P Liang, CD Manning, Learning language games through interaction in *Proceedings in the 2016 annual meeting*

of the association for computational linguistics. (2016).
101. GP Laput, et al., Pixeltone: A multimodal interface for image editing in *Proceedings of the SIGCHI Conference on Human Factors in Computing Systems.* (ACM), pp. 2185–2194 (2013).
102. X Rong, S Yan, S Oney, M Dontcheva, E Adar, Codemend: Assisting interactive programming with bimodal embedding in *Proceedings of the 29th Annual Symposium on User Interface Software and Technology.* (ACM), pp. 247–258 (2016).
103. J Weizenbaum, , et al., Eliza—a computer program for the study of natural language communication between man and machine. *Commun. ACM* **9**, 36–45 (1966).
104. T Winograd, F Flores, FF Flores, *Understanding computers and cognition: A new foundation for design.* (Intellect Books), (1986).
105. E Fast, B Chen, J Mendelsohn, J Bassen, MS Bernstein, Iris: A conversational agent for complex tasks in *Proceedings of the 2018 CHI Conference on Human Factors in Computing Systems.* (ACM), p. 473 (2018).
106. TH Wen, et al., Multi-domain neural network language generation for spoken dialogue systems in *Proceedings of the 2016 Conference of the North American Chapter of the Association for Computational Linguistics: Human Language Technologies.* (2016).
107. T Zhao, K Lee, M Eskenazi, Dialport: Connecting the spoken dialog research community to real user data in *IEEE Spoken Language Technology Workshop (SLT).* (IEEE), pp. 83–90 (2016).
108. RE Banchs, H Li, Iris: a chat-oriented dialogue system based on the vector space model in *Proceedings of the ACL 2012 System Demonstrations.* (Association for Computational Linguistics), pp. 37–42 (2012).
109. A Ritter, C Cherry, WB Dolan, Data-driven response generation in social media in *Proceedings of the conference on empirical methods in natural language processing.* (Association for Computational Linguistics), pp. 583–593 (2011).
110. J Li, et al., A persona-based neural conversation model (2016).
111. RJL John, N Potti, JM Patel, Ava: From data to insights through conversation in *CIDR.* (2017).
112. G Campagna, R Ramesh, S Xu, M Fischer, MS Lam, Almond: The architecture of an open, crowdsourced, privacy-preserving, programmable virtual assistant in *Proceedings of the 26th International Conference on World Wide Web.* (International World Wide Web Conferences Steering Committee), pp. 341–350 (2017).
113. J Hempel, Facebook launches m, its bold answer to siri and cortana. *Wired. Retrieved January* **1**, 2017 (2015).
114. D Bohus, AI Rudnicky, The ravenclaw dialog management framework: Architecture and systems. *Comput. Speech & Lang.* **23**, 332–361 (2009).
115. J Cheng, MS Bernstein, Flock: Hybrid crowd-machine learning classifiers in *Proceedings of the 18th ACM conference on computer supported cooperative work & social computing.* (ACM), pp. 600–611 (2015).
116. JC Chang, A Kittur, N Hahn, Alloy: Clustering with crowds and computation in *Proceedings of the 2016 CHI Conference on Human Factors in Computing Systems.* (ACM), pp. 3180–3191 (2016).
117. J Allen, et al., Plow: A collaborative task learning agent in *AAAI.* Vol. 7, pp. 1514–1519 (2007).
118. LA Adamic, J Zhang, E Bakshy, MS Ackerman, Knowledge sharing and yahoo answers: everyone knows something in *Proceedings of the 17th international conference on World Wide Web.* pp. 665–674 (2008).
119. G Wang, K Gill, M Mohanlal, H Zheng, BY Zhao, Wisdom in the social crowd: an analysis of quora in *Proceedings of the 22nd international conference on World Wide Web.* pp. 1341–1352 (2013).
120. MR Morris, J Teevan, K Panovich, What do people ask their social networks, and why? a survey study of status message q&a behavior in *Proceedings of the SIGCHI conference on Human factors in computing systems.* pp. 1739–1748 (2010).
121. J Nichols, JH Kang, Asking questions of targeted strangers on social networks in *Proceedings of the ACM 2012 conference on Computer Supported Cooperative Work.* pp. 999–1002 (2012).
122. J Park, R Krishna, P Khadpe, L Fei-Fei, M Bernstein, Ai-based request augmentation to increase crowdsourcing participation in *Proceedings of the AAAI Conference on Human Computation and Crowdsourcing.* Vol. 7, pp. 115–124 (2019).
123. E Agichtein, C Castillo, D Donato, A Gionis, G Mishne, Finding high-quality content in social media in *Proceedings of the 2008 international conference on web search and data mining.* pp. 183–194 (2008).
124. Y Liu, J Bian, E Agichtein, Predicting information seeker satisfaction in community question answering in *Proceedings of the 31st annual international ACM SIGIR conference on Research and development in information retrieval.* pp. 483–490 (2008).
125. C Lampe, J Vitak, R Gray, N Ellison, Perceptions of facebook's value as an information source in *Proceedings of the SIGCHI conference on human factors in computing systems.* pp. 3195–3204 (2012).
126. J Bian, Y Liu, E Agichtein, H Zha, Finding the right facts in the crowd: factoid question answering over social media in *Proceedings of the 17th international conference on World Wide Web.* pp. 467–476 (2008).
127. M Surdeanu, M Ciaramita, H Zaragoza, Learning to rank answers on large online qa collections in *Proceedings of ACL-08: HLT.* pp. 719–727 (2008).
128. K Ling, et al., Using social psychology to motivate contributions to online communities. *J. Comput. Commun.* **10**, 00–00 (2005).
129. RB Cialdini, RB Cialdini, Influence: The psychology of persuasion (1993).
130. M Bouguessa, B Dumoulin, S Wang, Identifying authoritative actors in question-answering forums: the case of yahoo! answers in *Proceedings of the 14th ACM SIGKDD international conference on Knowledge discovery and data mining.* pp. 866–874 (2008).

131. K He, X Zhang, S Ren, J Sun, Deep residual learning for image recognition in *Proceedings of the IEEE conference on computer vision and pattern recognition.* pp. 770–778 (2016).
132. P Rajpurkar, J Zhang, K Lopyrev, P Liang, Squad: 100,000+ questions for machine comprehension of text in *Proceedings of the 2016 Conference on Empirical Methods in Natural Language Processing.* (2016).
133. V Lai, et al., Human-ai collaboration via conditional delegation: A case study of content moderation in *Proceedings of the SIGCHI conference on Human factors in computing systems.* (2022).
134. D Horowitz, SD Kamvar, The anatomy of a large-scale social search engine in *Proceedings of the 19th international conference on World wide web.* pp. 431–440 (2010).
135. H Rashkin, EM Smith, M Li, YL Boureau, Towards empathetic open-domain conversation models: A new benchmark and dataset in *ACL.* (2019).
136. O Russakovsky, et al., Imagenet large scale visual recognition challenge. *Int. journal computer vision* **115**, 211–252 (2015).
137. T Shen, A Kar, S Fidler, Learning to caption images through a lifetime by asking questions in *Proceedings of the IEEE International Conference on Computer Vision.* pp. 10393–10402 (2019).
138. M Lewis, D Yarats, Y Dauphin, D Parikh, D Batra, Deal or no deal? end-to-end learning of negotiation dialogues in *Proceedings of the 2017 Conference on Empirical Methods in Natural Language Processing.* pp. 2443–2453 (2017).
139. A Razavi, A van den Oord, O Vinyals, Generating diverse high-fidelity images with vq-vae-2 in *Advances in neural information processing systems.* pp. 14866–14876 (2019).
140. P Khadpe, R Krishna, L Fei-Fei, J Hancock, M Bernstein, Conceptual metaphors impact perceptions of human-ai collaboration in *Proceedings of the 23th ACM conference on computer supported cooperative work & social computing.* (2020).
141. J Buolamwini, T Gebru, Gender shades: Intersectional accuracy disparities in commercial gender classification in *Conference on fairness, accountability and transparency.* pp. 77–91 (2018).
142. O Solon, Facial recognition's 'dirty little secret': Millions of online photos scraped without consent. *NBC News, March* **12** (2019).
143. M Murgia, Who's using your face? the ugly truth about facial recognition (2019).
144. ST Roberts, "commercial content moderation: Digital laborers' dirty work. (2016).
145. ME Whiting, G Hugh, MS Bernstein, Fair work: Crowd work minimum wage with one line of code in *Proceedings of the AAAI Conference on Human Computation and Crowdsourcing.* Vol. 7, pp. 197–206 (2019).
146. TY Lin, et al., Microsoft coco: Common objects in context in *European conference on computer vision.* (Springer), pp. 740–755 (2014).
147. MA DeVito, D Gergle, J Birnholtz, "algorithms ruin everything" # riptwitter, folk theories, and resistance to algorithmic change in social media in *Proceedings of the 2017 CHI conference on human factors in computing systems.* pp. 3163–3174 (2017).
148. J Angwin, J Larson, S Mattu, L Kirchner, Machine bias. *ProPublica, May* **23**, 2016 (2016).
149. B Liu, et al., When machine learning meets privacy: A survey and outlook. *ACM Comput. Surv. (CSUR)* **54**, 1–36 (2021).
150. Y Gu, Y Yao, W Liu, J Song, We know where you are: Home location identification in location-based social networks in *2016 25th International Conference on Computer Communication and Networks (ICCCN).* (IEEE), pp. 1–9 (2016).
151. AC Squicciarini, C Caragea, R Balakavi, Analyzing images' privacy for the modern web in *Proceedings of the 25th ACM conference on Hypertext and social media.* pp. 136–147 (2014).
152. S Zerr, S Siersdorfer, J Hare, E Demidova, Privacy-aware image classification and search in *Proceedings of the 35th international ACM SIGIR conference on Research and development in information retrieval.* pp. 35–44 (2012).
153. J Mahmud, J Nichols, C Drews, Home location identification of twitter users. *ACM Transactions on Intell. Syst. Technol. (TIST)* **5**, 1–21 (2014).
154. W Meng, X Xing, A Sheth, U Weinsberg, W Lee, Your online interests: Pwned! a pollution attack against targeted advertising in *Proceedings of the 2014 ACM SIGSAC Conference on Computer and Communications Security.* pp. 129–140 (2014).
155. A Reznichenko, P Francis, Private-by-design advertising meets the real world in *Proceedings of the 2014 ACM SIGSAC Conference on Computer and Communications Security.* pp. 116–128 (2014).
156. J Yu, B Zhang, Z Kuang, D Lin, J Fan, iprivacy: image privacy protection by identifying sensitive objects via deep multi-task learning. *IEEE Transactions on Inf. Forensics Secur.* **12**, 1005–1016 (2016).
157. T Orekondy, B Schiele, M Fritz, Towards a visual privacy advisor: Understanding and predicting privacy risks in images in *Proceedings of the IEEE international conference on computer vision.* pp. 3686–3695 (2017).
158. SCS Cheung, H Wildfeuer, M Nikkhah, X Zhu, W Tan, Learning sensitive images using generative models in *2018 25th IEEE International Conference on Image Processing (ICIP).* (IEEE), pp. 4128–4132 (2018).
159. T Orekondy, M Fritz, B Schiele, Connecting pixels to privacy and utility: Automatic redaction of private information in images in *Proceedings of the IEEE Conference on Computer Vision and Pattern Recognition.* pp. 8466–8475 (2018).
160. Q Sun, et al., Natural and effective obfuscation by head inpainting in *Proceedings of the IEEE Conference on Computer Vision and Pattern Recognition.* pp. 5050–5059 (2018).
161. A Triastcyn, B Faltings, Generating artificial data for private deep learning (2018).

162. A Jahanian, X Puig, Y Tian, P Isola, Generative models as a data source for multiview representation learning (2021).
163. AD Kramer, JE Guillory, JT Hancock, Experimental evidence of massive-scale emotional contagion through social networks. *Proc. Natl. Acad. Sci.* **111**, 8788–8790 (2014).
164. J Cheng, M Bernstein, C Danescu-Niculescu-Mizil, J Leskovec, Anyone can become a troll: Causes of trolling behavior in online discussions in *Proceedings of the 2017 ACM conference on computer supported cooperative work and social computing.* (ACM), pp. 1217–1230 (2017).
165. M Jakesch, M French, X Ma, JT Hancock, M Naaman, Ai-mediated communication: How the perception that profile text was written by ai affects trustworthiness in *Proceedings of the 2019 CHI Conference on Human Factors in Computing Systems.* pp. 1–13 (2019).
166. D Silver, et al., Mastering the game of go with deep neural networks and tree search. *nature* **529**, 484 (2016).
167. D Silver, et al., Mastering the game of go without human knowledge. *nature* **550**, 354–359 (2017).
168. R Campa, The rise of social robots: a review of the recent literature. *J. Evol. Technol.* **26** (2016).
169. CB Frey, MA Osborne, The future of employment: How susceptible are jobs to computerisation? *Technol. forecasting social change* **114**, 254–280 (2017).
170. M Webb, The impact of artificial intelligence on the labor market (2019).
171. D Autor, D Mindell, EB Reynolds, The work of the future: Shaping technology and institutions (2019).
172. S Stumpf, et al., Toward harnessing user feedback for machine learning in *Proceedings of the 12th international conference on Intelligent user interfaces.* (ACM), pp. 82–91 (2007).
173. V Chidambaram, YH Chiang, B Mutlu, Designing persuasive robots: how robots might persuade people using vocal and nonverbal cues in *Proceedings of the seventh annual ACM/IEEE international conference on Human-Robot Interaction.* (ACM), pp. 293–300 (2012).
174. T Kulesza, S Stumpf, M Burnett, I Kwan, Tell me more?: the effects of mental model soundness on personalizing an intelligent agent in *Proceedings of the SIGCHI Conference on Human Factors in Computing Systems.* (ACM), pp. 1–10 (2012).
175. AM Rashid, et al., Motivating participation by displaying the value of contribution in *Proceedings of the SIGCHI conference on Human Factors in computing systems.* (ACM), pp. 955–958 (2006).
176. A Das, et al., Visual dialog in *Proceedings of the IEEE Conference on Computer Vision and Pattern Recognition.* pp. 326–335 (2017).
177. J Deng, J Krause, L Fei-Fei, Fine-grained crowdsourcing for fine-grained recognition in *Proceedings of the IEEE conference on computer vision and pattern recognition.* pp. 580–587 (2013).
178. E Hunt, Tay, microsoft's ai chatbot, gets a crash course in racism from twitter. *The Guard.* **24** (2016).
179. G Neff, P Nagy, Talking to bots: Symbiotic agency and the case of Tay. *Int. J. Commun.* **10**, 17 (2016).
180. J Li, AH Miller, S Chopra, M Ranzato, J Weston, Learning through dialogue interactions by asking questions in *International Conference on Learning and Representation.* (2017).
181. A See, S Roller, D Kiela, J Weston, What makes a good conversation? how controllable attributes affect human judgments in *NAACL.* (2019).
182. A Radford, et al., Learning transferable visual models from natural language supervision in *Proceedings of ICML.* (2021).
183. S Zhou*, et al., Hype: A benchmark for human eye perceptual evaluation of generative models in *NeurIPS.* (2019).
